# Spectroscopic, docking, antiproliferative, and anticancer activity of novel metal derivatives of phenylacetohydrazide Schiff base on different human cancer cell lines

**DOI:** 10.1186/s13065-025-01417-1

**Published:** 2025-03-15

**Authors:** Manal A. Afifi, Anas A. Rasmy, Emad M. Elzayat, Samir M. El-Medani, Mohamed R. Shehata, Fatma M. Elantabli

**Affiliations:** 1 Chemistry Department, Faculty of Science, El-Fayoum University, El-Fayoum, Egypt; 2 Department of Biotechnology, Faculty of Sciences, Cairo University, Giza, Egypt; 3 Chemistry Department, Faculty of Science, Cairo University, Giza, Egypt

**Keywords:** Phenylacetohydrazide Schiff base, Metal complexes, Anticancer exploration, Molecular docking simulation, DFT

## Abstract

In the last two decades, many Schiff bases have been investigated due to the importance of their metal complexes in the medical field and drug industry. The Schiff base metal complexes have several applications as anticancer agents because they have a high binding ability to nucleic acids (DNA and RNA). The Schiff base H_2_L, derived from the condensation of 2-phenylacetohydrazide and 2-hydroxynaphthaldehyde, was reacted with Fe^2+^, Zn^2+^, Cd^2+^, and Pt^2+^ to form the unique metal complexes [Fe(HL)_2_], [Zn_2_(HL)_2_](CH_3_COO)_2_, [Cd_2_(HL)_2_](CH_3_COO)_2_, and [Pt(H_2_L)Cl_2_]. Various analytical and spectroscopic techniques were used to characterize the newly reported compounds. The elemental and spectroscopic analysis revealed that the platinum complex was a square planar with 2.5 water molecules in the crystal lattice, whereas the iron complex had an octahedral geometry. The thermogravimetric analysis demonstrated the stability of the complexes and validated the dimerization of zinc and cadmium complexes. DFT calculations were investigated to obtain the optimized structure of the ligand and its complexes. Biological screening and molecular docking studies of the ligand and complexes were reported to explore their potential application as therapeutic drugs. Among the tested complexes, [Cd_2_(HL)_2_](CH_3_COO)_2_ complex showed the best cytotoxic effect, especially on the human colorectal cancer cell line (HCT116, IC_50_ = 0.329 µg/ml) as compared to normal human skin fibroblast (HSF, IC_50_ = 5.240 µg/ml) and selectivity index (SI) = 15.93. It represents a promising anticancer drug compared to Cisplatin (IC_50_ = 2.25µg/ml, SI = 4.92). The biological studies and molecular docking were correlated to each other.

**Supplementary Information:**

The online version contains supplementary material available at 10.1186/s13065-025-01417-1.

## Introduction

Researchers have been interested in developing new anticancer drugs with better action and high efficiency over decades [1]. Schiff base ligands and their metal complexes are suitable and effective compounds for anticancer drugs [2–7]. They have mutable coordination sites, such as N, ONNO, NN, and ONO [8, 9]. Schiff base ligands can easily form stable complexes with most transition metals [10]. The interest in the Schiff base ligands and their metal complexes lies in their different and important applications such as anticancer, anti-inflammatory, antiradical, antimicrobial, anti-HIV, antibacterial, antitubercular, antitumor, antifungal, and DNA cleavage [11–14]. There are also wide uses for the Schiff base complexes such as in medicine, drugs, chemical analysis, industrial catalysis, photochromism, and metal corrosion [15, 16]. The Schiff base derivatives, and their metal complexes, were initiated to have vital applications in therapeutic medicine and pharmacology [17, 18]. They could be used as drugs to treat many diseases, such as cancer maladies, and microbial, fungal, and viral infections [19]. Diversity in applications of Schiff base complexes may be due to their synthetic flexibility, high thermal stability, and stability under various reductive and oxidative agent conditions [20, 21]. The Schiff base metal complexes have a high ability to bind to nucleic acids (DNA and RNA). This explains the several applications of these complexes as anticancer, anti-inflammatory, and antimicrobial and explains the importance of these complexes to be used as drugs for treating many diseases like cancer, fever, AIDS, malaria, Alzheimer's disease, fungal infections, and diabetes [22].

Hydrazides and hydrazones and their derivatives, have biological effects and also revealed high anticancer, anti-inflammatory, antimicrobial, antibacterial, and antifungal applications in addition to high DNA binding activities [23–28]. Schiff bases derived from hydrazides and hydrazones for example isoniazid, benzohydrazide, pyridinehydrazone, and phenylacetohydrazide and their metal complexes, exposed antiviral activities and large antimicrobial [29–31]. They can act as a very decent probable for drugs used, in the treatment of genetic disorders like thalassemia [19, 32]. Fe(II), Pt(II), Zn(II), and Cd(II) Schiff base complexes were found to show anticancer, anti-inflammatory, antibacterial, and DNA-binding activities. MCF-7 cell line was used to examine the anticancer activity of a new Schiff base (HL) ligand of (E)-2-hydroxy-*N*′-((E)-1-(2-(*p*-tolyl)hydrazono)propan-2-ylidene) benzo hydrazide and its Pd(II) and Zn(II) complexes in vitro. The results showed that Zn(II) complex is the most active anticancer drug as it prompted a very similar genotoxic effect as Cisplatin with a very low IC_50_ value [33]. Three complexes [Pd(AMI)Cl_2_](1), [Cu(AMI)L_1_](2), and [Cu(AMI)L_2_·2H_2_O](3) where AMI = amino methyl imidazole, L_1_ = oxalate and L_2_ = malonate, were synthesized and characterized. Complex two verified good anticancer activity against MCF-7 and Hela cells. The results of the DPPH radical scavenging assay recognized that complex one was a more powerful antioxidant than other complexes. Antimicrobial assays recognized complex three as the most active species in this study^.^ [34]. Transition metal complexes (Fe, Zn, Cd, Ag, Pd) of 2-hydroxybenzylidene-2-phenylacetohydrazide were previously synthesized and exhibited high biocidal activity, which encouraged us to complete our progress on this class of ligands [5].

In the continuity of our previous study of the hydrazones as ligands in complexes with Co(II), Ni(II), and Cu(II), [11] and according to the several important applications of hydrazones complexes, we here continue the study of *N*ʹ-((1-hydroxynaphthalen-2-yl)methylene)-2-phenylacetohydrazide ligand with some other transition metals namely Fe(II), Pt(II), Zn(II) and Cd(II). The spectroscopic studies and thermal stability of the new complexes as well as the anticancer activities of the complexes against different cell lines were studied.

## Experimental

### Materials

Fe(ClO_4_)_2_, K_2_[PtCl_4_], Zn(CH_3_COO)_2_, Cd(CH_3_COO)_2_, 2-hydroxynaphthaldehyde, were purchased from Sigma-Aldrich Chemie (Germany). Ethyl acetate, DMF, and ethanol solvents were of analytical reagent grade and used without purification.

### Instrumentation

IR measurements (KBr pellets) were performed on a Cary 630 FTIR Spectrometer. ^1^H-NMR spectra were carried out on a Varian Unity 500 MHz spectrometer at 298 K. Magnetic susceptibilities of the paramagnetic complexes in the solid state (Gouy method) were carried out on a Sherwood Scientific magnetic susceptibility balance. Elemental analyses were performed on a Perkin-Elmer 2400 CHN elemental analyzer. Conductivity measurements (1 × 10^–3^ M in DMF at 25 °C) were measured using the Jenway 4010 conductivity meter. Thermogravimetric analysis (TGA and DTG) was performed under a nitrogen atmosphere with a heating rate of 10 °C/min using a Schimadzu DT-50 thermal analyzer. Mass spectra (EIS, 70 eV) were carried out with a Finnigan MAT SSQ 7000.

spectrometer at Cairo University. Electron paramagnetic resonance (EPR) spectra were prepared at Elexsys, E500, Bruker Company. Bruker Bio Spin GmbH was used to obtain the electron spin resonance spectra at 25 °C in the X-band frequency of 9.714 GHz and on a microcrystalline powder with a microwave power of 2.012 mW. UV–Vis spectra were carried out on a DS5-Edinburgh spectrophotometer.

### Synthesis of the ligand and complexes

#### Synthesis of *N*ʹ-((2-hydroxynaphthalen-1-yl)methylene)-2-phenylacetohydrazide (H_2_L)

The H_2_L ligand was synthesized as reported in the literature with a slight modification [12].^.^ Typically, a mixture of 2-phenylacetohydrazide (1.50 g, 10 mmol) and 2-hydroxy naphthaldehyde (1.72 g, 10 mmol) in ethyl acetate was heated to reflux for 4 h. Evaporation of the solvent gave a fluffy yellow solid. The residue was left to dry overnight under vacuum. Washing with diethyl ether several times yielded a yellow solid. The solid was recrystallized from ethyl acetate to give 2.59 mg, Yield 75%, m.p. 210 − 213 °C. ΛM (Ω^−1^ mol^−1^ cm^−2^) 4.0. Anal. Calc. (%): C, 74.98; H, 5.30; N, 9.21, (found): C, 74.77; H, 5.20; N, 9.12. FTIR; υ/cm^−1^: 3435 (OH), 3199 (NH), 1666 (C=O), 1623 (C=N_azomethine_), 1270 (C–O), 1077(N–N); ^1^HNMR, ppm: 12.51 (s, 1H, OH-keto), 11.85, 11.35 (s, 1H, OH-enol), 10.00 (s, 1H, NH), 9.22 (s, 1H, CH=N-keto), 8.91 (s, 1H, CH=N-enol), 7.18–8.25 (m, 10H, aromatic), 4.00 (s, 2H, CH_2_- keto), 3.62 (s, 2H, CH_2_-enol).

#### ***Synthesis of ferrous complex; [Fe(HL)***_***2***_***]***

A mixture of ferrous perchlorate (1mmol, 254 mg) and the ligand (1mmol, 304 mg) was separately dissolved in ethanol and ethyl acetate, respectively, and then heated to reflux for 4 h. The mixture immediately gave a dark green color. The mixture was left for two days to precipitate a green solid. The solid was washed with diethyl ether several times and left to dry. Yield 79%, m. p. over 350°C. ΛM (Ω^−1^mol^−1^cm^−2^) 12.8. μ_eff_ (B.M.) 4.68. Anal. Calc. (%): C, 68.89; H, 4.56; N, 8.46, (found): C, 68.75; H, 4.23; N, 8.34 (Table 1). FTIR; υ/cm^−1^: 3210 (NH), 1596 (C=O), 1569 (C=N)_azomethine_, 1191 (C–O).

**Table 1:** Physical and analytical data of the H_2_L ligand and its complexes

Compound	Color	Y (%)	Elemental analysis, Found (calc.)			Molecular weight	μ_eff_ (BM)	Λ (ohm^−1^ mol^−1^ cm^2^)
			% C	% H	% N	M.M		
H_2_L (C_19_H_16_N_2_O_2_)	Yellow	75	74.77 (74.98)	5.2 (5.30)	9.12 (9.21)	304.348	–	–
[Fe(HL)_2_] (FeC_38_H_30_N_4_O_4_)	Dark green	79	68.75 (68.89)	4.23 (4.56)	8.34 (8.46)	662.51	4.68	12.8
[Zn_2_(HL)_2_](CH_3_COO)_2_ (Zn_2_C_42_H_36_N_4_O_8_)	Pale yellow	91	58.78 (58.96)	4.21 (4.24)	6.35 (6.55)	855.52	–	22
[Cd_2_(HL)_2_](CH_3_COO)_2_ (Cd_2_C_42_H_36_N_4_O_8_)	Yellow	91	53.09 (53.12)	3.66 (3.82)	5.77 (5.90)	949.58	–	21
[Pt(H_2_L)Cl_2_]. 2.5H_2_O (PtC_19_H_21_N_2_O_4.5_Cl_2_)	Yellow	59	37.04 (37.08)	3.32 (3.44)	4.50 (4.55)	615.37	–	4.8

#### ***Synthesis of zinc complex; [Zn***_***2***_***(HL)***_***2***_***](CH***_***3***_***COO)***_***2***_

A mixture of zinc acetate dihydrate (1mmol, 219 mg) and the ligand (1mmol, 304 mg) was separately dissolved in ethanol and ethyl acetate, respectively, then heated to reflux for 4 h. The mixture gave a pale-yellow color immediately. The yellow solid complexes formed were isolated by filtration. The solid was washed with diethyl ether several times and left to dry. Yield 91%, m. p. over 350 °C. Anal. Calc. (%): C, 58.96; H, 4.24; N, 6.55, (found): C, 58.78; H, 4.21; N, 6.35. (FTIR; υ/cm^−1^): 3425 (OH), 1613, 1542 (C=N_azomethine_), 1249 (C-O). ^1^HNMR (DMSO, ppm): 11.00(s, 2H, OH), 9.43 (s, 2H, CH=N), 8.03–6.65 (m, 22H_aromatic_), 3.62 (d, 2H, CH_2_), 3.56 (d, 2H, CH_2_), 1.87 (s, 6H, 2CH_3_). ΛM (Ω^−1^mol^−1^cm^−2^) 22.

#### ***Synthesis of cadmium complex; [Cd***_***2***_***(HL)***_***2***_***](CH***_***3***_***COO)***_***2***_

A mixture of cadmium acetate dihydrate (1mmol, 266 mg) and the ligand (1mmol, 304 mg) was dissolved in ethanol and ethyl acetate and then heated to reflux for 4 h. The mixture gave a yellow color immediately. The yellow solid complex was isolated by filtration. The solid was washed with diethyl ether several times and left to dry. Yield 91%, m. p. over 350 °C. Anal. Calc. (%): C, 53.12; H, 3.82; N, 5.90, (found): C, 53.09; H, 3.66; N, 5.77. FTIR; υ/cm^−1^: 3426 (OH), 1618, 1544 (C=N), 1190 (C–O);^1^HNMR (DMSO, ppm):10.98 (s, 2H, OH) 9.27 (s, 2H, CH=N), 7.89–6.80 (m, 22H_aromatic_), 3.66 (s, 4H, 2CH_2_), 1.85 (s, 6H, 2CH_3_). ΛM (Ω^−1^mol^−1^cm^−2^) 21.

#### ***Synthesis of platinum complex; [Pt(H***_***2***_***L)Cl***_***2***_***]. 2.5H***_***2***_***O***

A solution of potassium tetrachloroplatinate (II) in deionized water (1mmol, 415 mg) and the ethanol solution of the ligand (1mmol, 304 mg) was mixed and heated to reflux for 4 h. The mixture gave a yellow color immediately. The mixture was left for one week at 4 °C to give a yellow solid. The solid was washed with diethyl ether several times and left to dry. Yield 59%, m. p. over 350 °C. Anal. Calc. (%): C, 37.08; H, 3.44; N, 4.55, (found): C, 37.04; H, 3.32; N, 4.50. FTIR; υ/cm^−1^: 3430 (OH), 1608, 1542 (C=N)_azomethine_, 1177 (C-O); ^1^HNMR (DMSO, ppm): 12.89(s, 1H, OH), 10.00 (s, 1H, OH), 9.51 (s, 1H, CH=N), 8.56–7.29 (m, 11H_aromatic_), 3.95 (s, 2H, CH_2_). ΛM (Ω^−1^mol^−1^cm^−2^) 4.8.

The physical properties of the ligand and the complexes are depicted in Table 1.

### Cell culture

Cells (HeLa, cervical cancer cells; MDA-MB-231, breast cancer cells; A375, skin cancer cells; A549, lung cancer cells; and HSF, normal skin fibroblasts cells) were supplied from Nawah Scientific (Cairo, Egypt). The cells were grown in DMEM medium supplemented with 10% fetal bovine serum (Gibco, Life Technologies Inc., UK) with 1% penicillin/streptomycin solution (Gibco). Cells were maintained at 37°C with 5% CO_2_.

### MTT assay

MTT in vitro cytotoxic assay assesses the metabolic activity of viable cells through the reduction of the water-soluble yellow dye by mitochondrial succinate dehydrogenase into the insoluble purple formazan [35]. In brief, cyanochalcone derivatives were dissolved in 100% dimethyl sulphoxide (DMSO, Serva, Heidelberg, Germany) and incubated with cells at the desired concentrations (0, 6.25, 12.5, 25, 50, 100, and 200 μM) for 48 h with the corresponding vehicle as control. 5-Florouracil (5FU) was used as a positive control. Following incubation, old media were discarded and MTT solution (50 μl, 0.5 mg/ml) was added. After 4 h of incubation, the insoluble formed formazan was dissolved in DMSO (100 μl) with a shaking plate for 15 min in a dark place. The absorbance readings were taken at 570 nm by an ELISA reader. Relative cell viability was calculated as a percentage of control untreated cells. The assay was done in four replicates. The dose–response curve was plotted using GraphPad Prism 8, and the values of IC_50_ (half-maximal inhibitory concentration) were determined using non-linear regression analysis. Finally, the selectivity index was calculated by dividing the IC_50_ for normal cells by the IC_50_ of cancer cells.

### Molecular docking studies

The molecular docking investigations were carried out using MOA2022 software [35], to find the possible binding ways of the most active site of the receptor of Methionine adenosyl-transferases in liver cancer (PDB ID: 5A19) [36].

### Molecular DFT studies

Density Functional Theory (DFT) calculations have been performed to elucidate the equilibrium geometry of the ligand and complexes at the B3LYP level of theory, where C, H, N, O, and Cl atoms at 6-311G^++^(dp) and metal atom at LANL2DZ using Gaussian 09 program [37, 38].

## Results and discussion

### Spectroscopic studies

#### IR spectroscopy

**Fig. 1:**
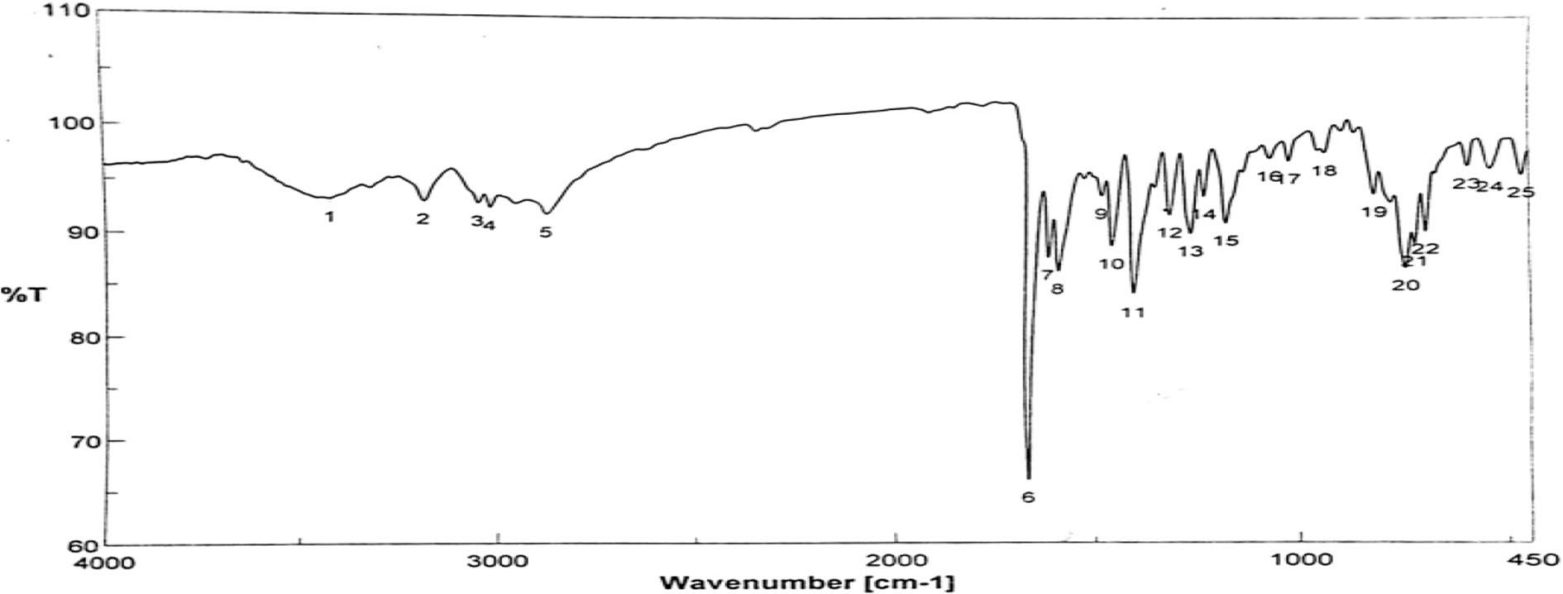
IR spectrum of H_2_L

**Scheme 1:**
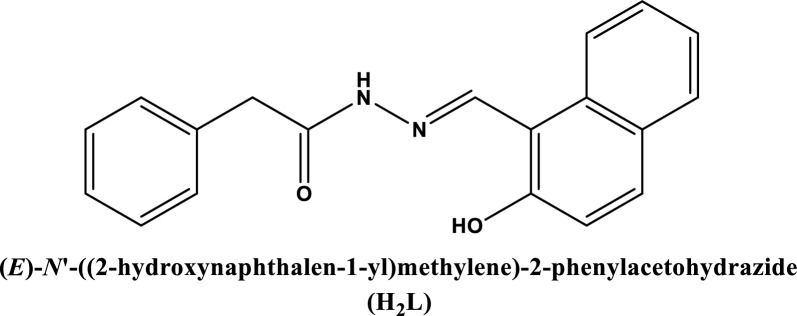
The structure of H_2_L ligand

**Fig. 2:**
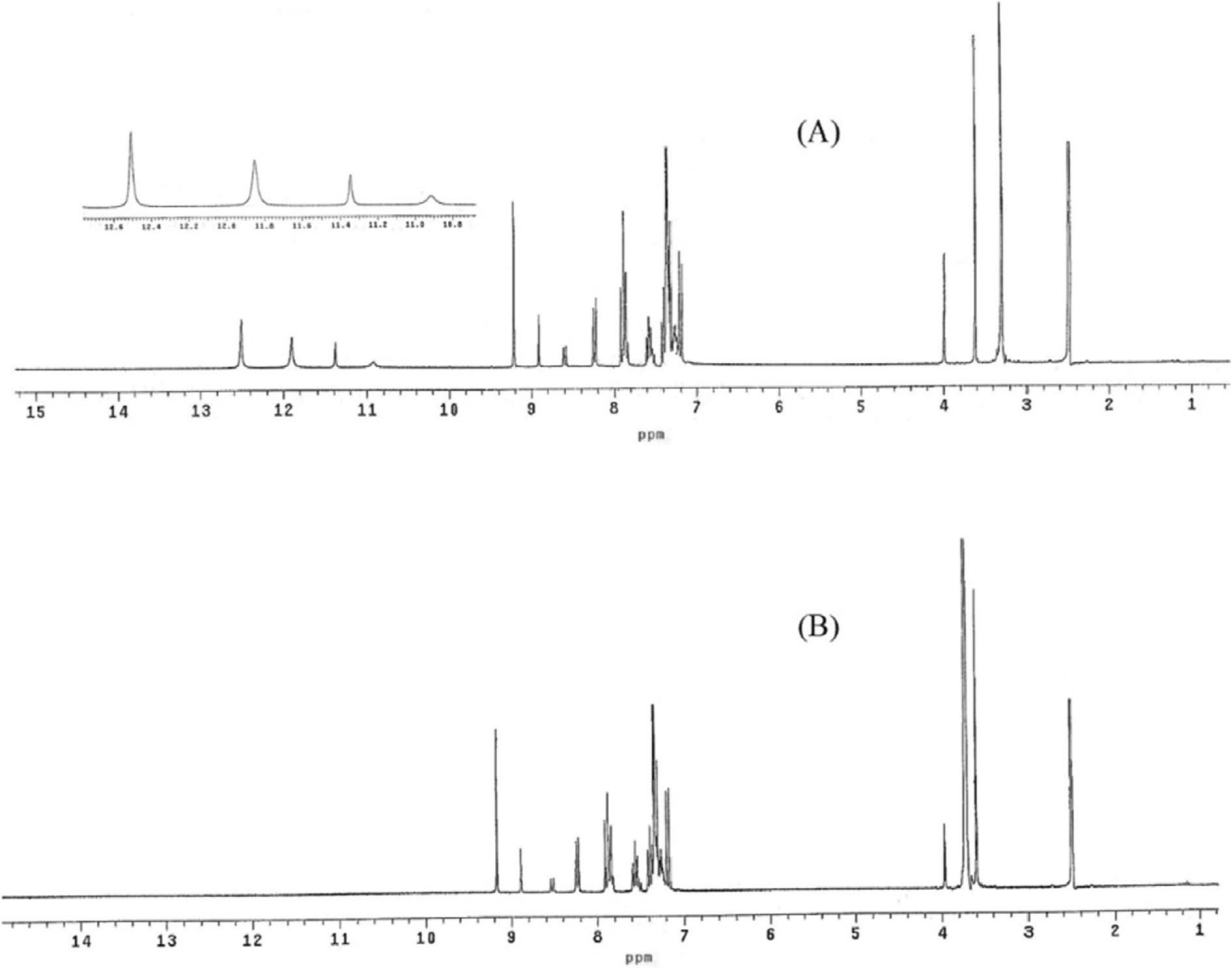
^1^HNMR of H_2_L ligand (**A**) in DMSO (**B**) in D_2_O

**Scheme 2:**
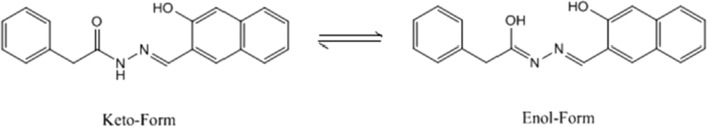
The enol and keto forms of H_2_L ligand

**Fig. 3:**
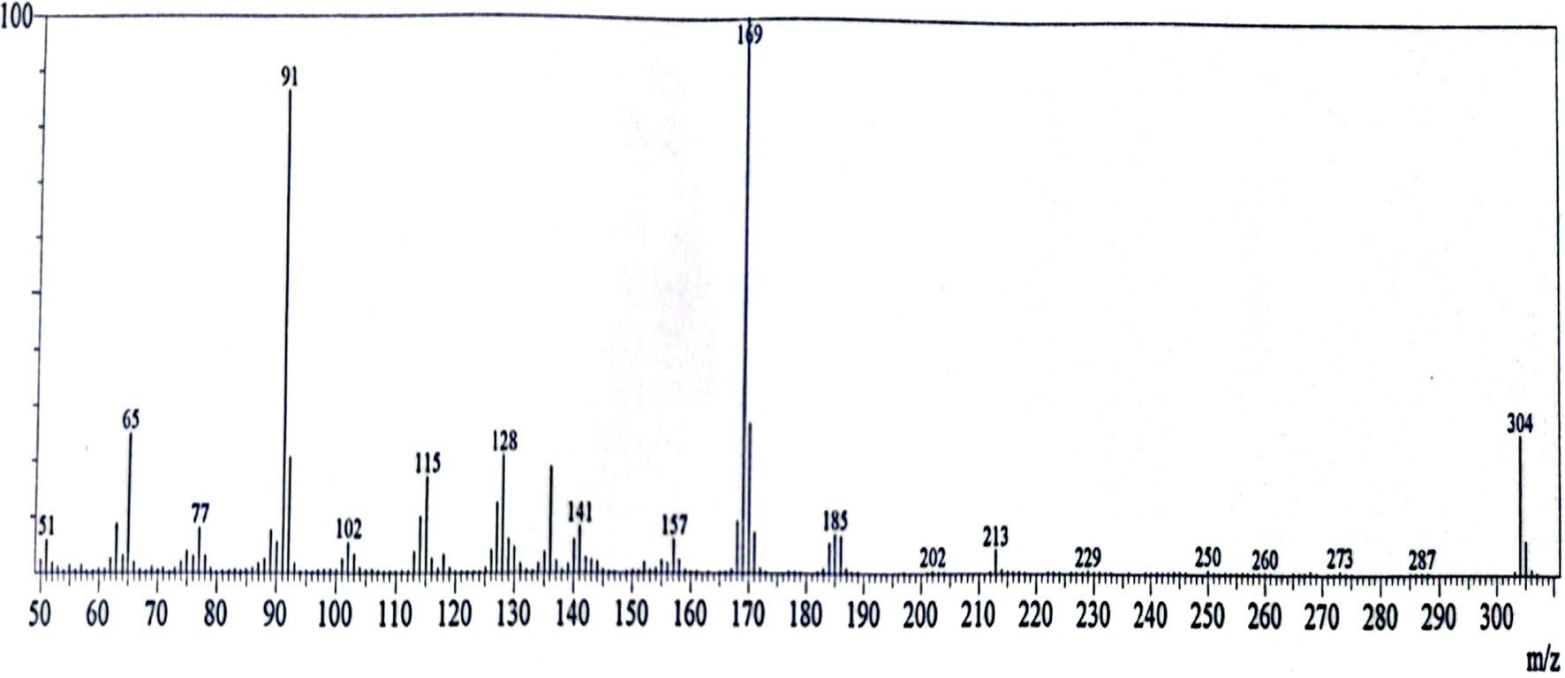
Mass spectrum of H_2_L ligand

The Schiff base ligand H_2_L was previously reported and synthesized by condensation of 2-hydroxynaphthaldehyde and 2-phenylacetohydrazide in ethyl acetate to yield *N*ʹ-((2-hydroxynaphthalen-1-yl)methylene)-2-phenylacetohydrazide [12]. The spectroscopic characterization (FTIR and ^1^H NMR) confirmed the structure of the ligand. The FTIR (Fig. 1) showed a broad band at 3435 cm^−1^ due to the stretching vibration of the hydroxyl group. Stretching vibrations at 3199, 1666, 1623, 1270 cm^−1^ were ascribed to ν(NH), ν(C=O), ν(CH=N), and ν(C–O), respectively [9, 39]. Thus, the IR confirmed the presence of the ligand in the solid state as a keto-form structure (Scheme 1). The ^1^H NMR spectrum of H_2_L in DMSO (Fig. 2A) showed a group of four broad singlets due to OH and NH protons at 12.51, 11.85, 11.35, and 10.91 ppm. When D_2_O was added, these signals vanished (Fig. 2B). The molecule is fluxional at the NMR time scale, as evidenced by the line broadening of the four signals at the same rate, which also proved the exchangeability of the OH and NH protons. According to Scheme 2, H2L was present in solution in two tautomeric (keto/enol) forms [5]. The mass spectrum of the ligand (Fig. 3) showed the parent peak m/z at 304 according to the molecular weight of the ligand.

**Scheme 3:**
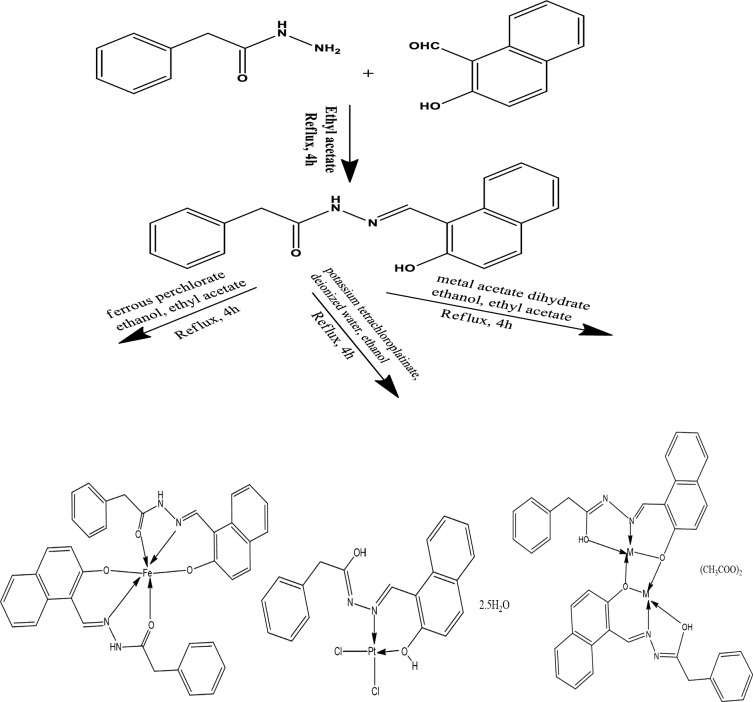
Schematic representation for the synthesis of H_2_L Schiff base ligand and its metal complexes (M=Zn or Cd)

**Fig. 4:**
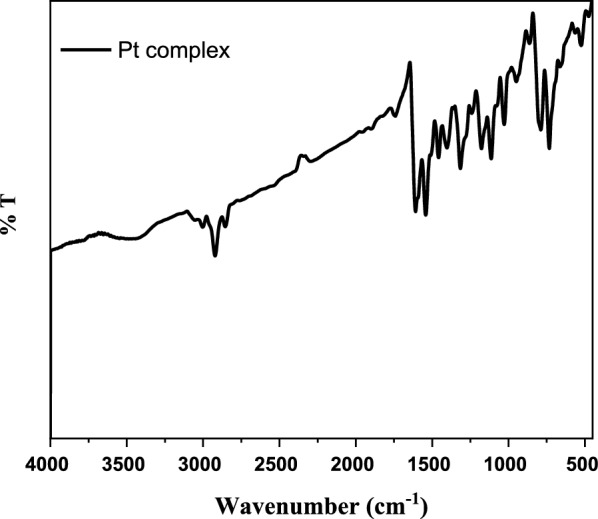
IR spectrum of the platinum complex

On account of the importance of metal Schiff base complexes in the medical field and drug industry, the reported Schiff base H_2_L was reacted with Fe^2+^, Zn^2+^, Cd^2+^, and Pt^2+^ to produce the novel metal complexes [Fe(HL)_2_], [Zn_2_(HL)_2_](CH_3_COO)_2_, [Cd_2_(HL)_2_](CH_3_COO)_2_, and [Pt(H_2_L)Cl_2_], respectively. In addition to elemental analyses, molar conductivity, magnetic measurements, and thermal analysis, various spectroscopic techniques, such as FT-IR, ^1^H-NMR, UV–vis, and Mass spectra were utilized to examine the predicted structures of the complexes. Table 1 depicts the physical properties and analytical data for the inspected complexes. According to elemental analysis and spectroscopic data, the proposed structures of the present complexes are shown in Scheme 3. The complexes' FT-IR spectra (Fig. 4 and Figs. S1–S3) confirmed the ligand's coordination sites that participated in the complex’s formation (Table 2).

**Table 2:** Important IR data of **H**_**2**_**L** ligand and its complexes

Compound	IR data, cm^−1^						
	ν(OH)	ν(NH)	ν(C=O)	ν(C=N)	ν(C–O)	ν(M–O)	ν(M–N)
H_2_L	3435 (b)	3199 (s)	1666 (vs)	1623 (m)	1270 (m)	–	–
[Fe(HL)_2_]	–	3210 (m)	1596 (s)	1569 (vs)	1191 (s)	519 (m)	477 (m)
[Zn_2_(HL)_2_](CH_3_COO)_2_	3425	–	–	1613 (s) 1542 (s)	1249 (s)	593(m)	439 (m)
[Cd_2_(HL)_2_](CH_3_COO)_2_	3426	–	–	1618(m) 1544 (s)	1190 (s)	544 (m)	459 (m)
[Pt(H_2_L)Cl_2_]. 2.5H_2_O	3430 (b)	–	–	1608(m) 1542(vs)	1177 (s)	527(m)	427 (m)

The IR spectrum of the iron complex displayed strong bands at 1596 and 1569 cm^−1^ according to stretching vibrations of (C=O) and (C=N) groups, respectively, with a significant shift from the ligand indicating the involvement of these function groups in coordination. Interestingly, a stretching band of the hydroxyl group has not appeared in the IR spectrum of the iron complex, however, the stretching frequency of (C–O) was shifted from the ligand (1270 cm^−1^) to (1191 cm^−1^) in the complex confirming the coordination of the deprotonated hydroxyl group to the metal center (Fig. S1). This result was confirmed by measuring the molar conductance which showed the neutral nature of the iron complex (see Table 1). These data emphasized the present Schiff base H_2_L acted as a tridentate ligand and coordinated with the ferrous ion through the oxygen of the carbonyl group, the nitrogen of the azomethine group, and the deprotonated hydroxyl group (Scheme 3). The elemental analysis of this complex confirmed the ligand was coordinated to the metal ion with the ratio 2 ligands: 1 metal to give octahedral structure geometry. In addition, non-ligand bands appeared at 519 cm^−1^ and 477 cm^−1^ referring to stretching frequencies of M–O and M–N bonds, respectively.

**Fig. 5:**
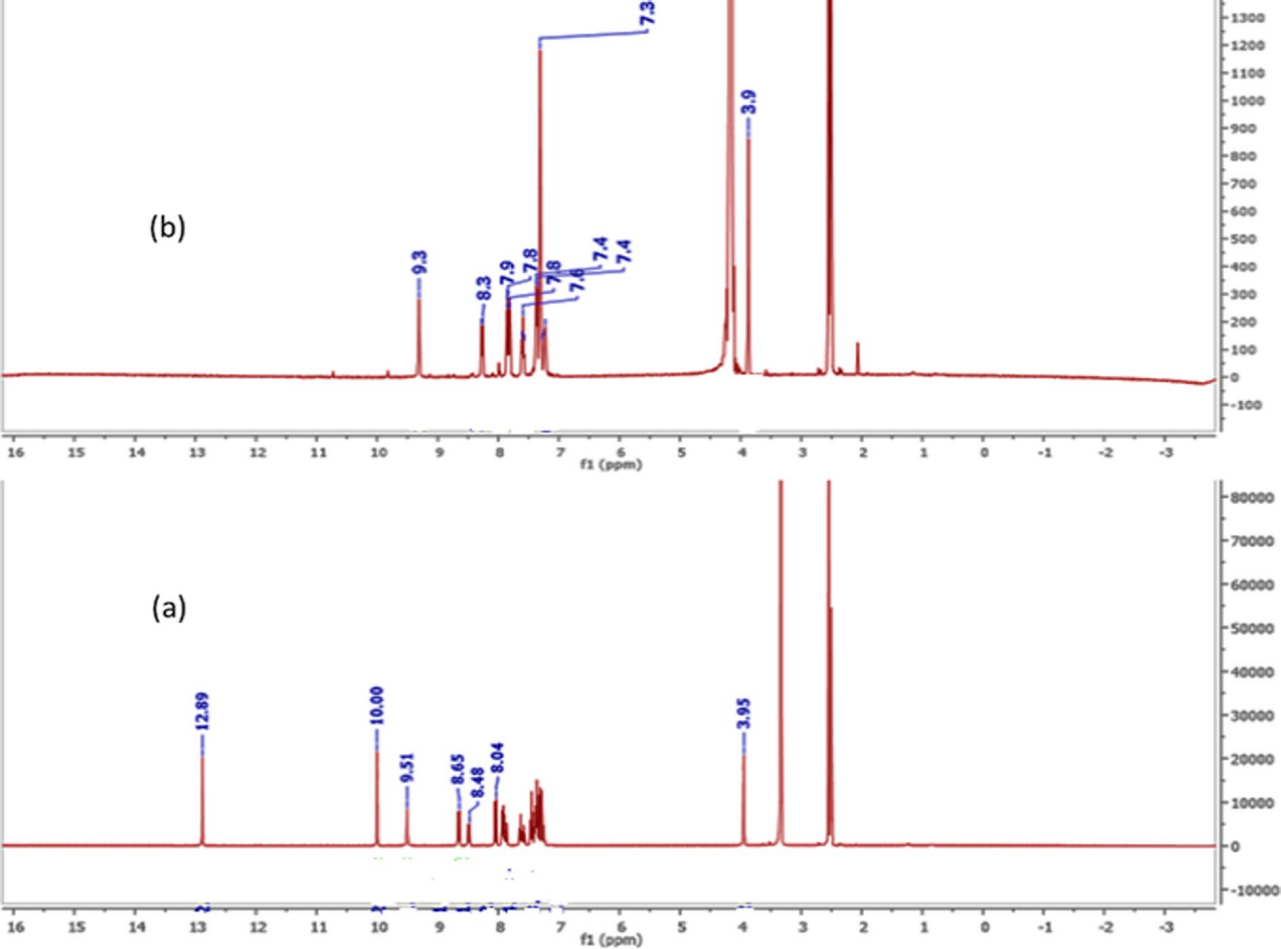
^1^HNMR of the platinum complex (**a**) in DMSO (**b**) in D_2_O

The IR spectra of the zinc and cadmium complexes displayed the same pattern. Stretching bands according to hydroxyl and azomethine groups have been shown in the IR spectra at 3425 and 1613 cm^−1^ with a proper shift from the ligand (3435 and 1623 cm^−1^) confirming the coordination of the ligands through these sites (Table 2). In addition, stretching frequencies at 544 and 459 cm^−1^ are due to ν(M–O) and ν(M–N), respectively (Figs. S2, S3). The elemental data proved the ligand was coordinated to the metal ion with a 1:1 ratio, however, the thermogravimetric analysis gave two moles of metal oxide as a residue in the cases of zinc and cadmium complexes. This finding asserted the dimerization of the two complexes which revealed that two ligands interacted with two metal ions [12]. The infrared spectrum of the platinum complex exhibited stretching frequencies for the υ(C=N) at 1608 and 1540 cm^−1^, respectively, with a proper shift from the ligand (Table 2, Fig. 4)). The stretching frequency of the hydroxyl group appeared in the IR spectrum around 3400 cm^−1^ indicating the involvement of the oxygen atom of the hydroxyl group without deprotonation on complexation. This conclusion was supported by the ^1^HNMR spectrum (Fig. 5, Table 3). The platinum atom was coordinated to the ligand with the hydroxyl group's oxygen, the azomethine group's nitrogen, and two chloride ligands to form a square planar structure. Similarly, the non-ligand bands appeared at 523 cm^−1^ and 427 cm^−1^ due to ν(M–O) and ν(M–N) bonds [40], respectively.

**Table 3:** The ^1^H NMR data for the H_2_L Schiff base and its complexes

Compound	^1^H NMR data, ppm
H_2_L	12.51 (s, 1H, OH_keto_), 11.85, 11.35 (s, 2H, OH_enol_), 10.00 (s, 1H, NH), 9.22 (s, 1H, CH=N_keto_), 8.91 (s, 1H, CH=N_enol_), 7.18–8. 25 (m, 10H, aromatic), 4.00 (s, 2H, CH_2keto_), 3.62 (s, 2H, CH_2enol_)
[Zn_2_(HL)_2_](CH3COO)_2_	9.43 (s, 2H, CH=N), 8.03–6.65 (m, 22H_aromatic_), 3.62 (d, 2H, CH_2_), 3.56 (d, 2H, CH_2_), 1.87 (s, 6H, 2CH_3_)
[Cd_2_(HL)_2_](CH3COO)_2_	9.27 (s, 2H, CH=N), 7.89–6.50(m, 22H_aromatic_), 3.66 (s, 4H, 2CH_2_), 1.85 (s, 6H, 2CH_3_)
[Pt(H_2_L)Cl_2_]. 2.5H_2_O	12.89 (s, 1H, OH), 10.00 (s, 1H, OH), 9.51 (s, 1 H, CH=N), 8.65–7.25 (m, 11H_aromatic_), 3.95 (s, 2H, CH_2_)

#### ^1^HNMR spectroscopy

**Scheme 4:**
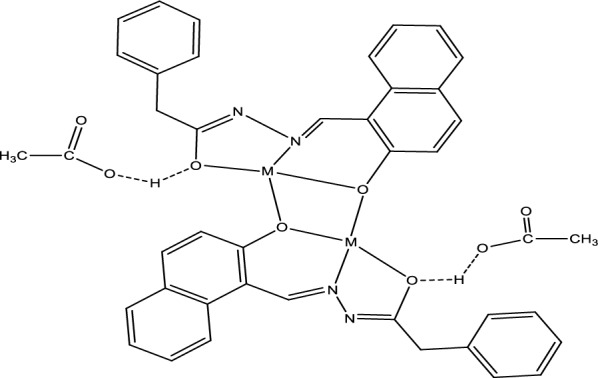
H-bond in Zn and Cd complexes (M=Zn or Cd)

The ^1^H NMR data confirmed the findings in the infrared spectra of the complexes (Table 3). The ^1^HNMR of zinc and cadmium complexes exhibited nearly the same pattern confirming the similarity of the geometry for the two complexes (Figs. S4, S5). Singlet signal at 9.43 and 9.27 ppm manifested in the proton ^1^HNMR spectra of Zn and Cd complexes, respectively, due to the proton of azomethine groups [41]. This signal was shifted from its value in the ligand (8.91 ppm) confirming the coordination of this group to the metal ion. Multiplets from 6.82 to 7.88 ppm appeared in the ^1^HNMR due to aromatic protons. Furthermore, the ^1^HNMR exhibited a singlet signal at 3.66 ppm due to methylene protons. A singlet signal valued at 1.85 ppm (6H) appeared in the ^1^HNMR due to the two methyl groups in two acetate counter ions. Interestingly, the ^1^H NMR spectrum of the zinc and cadmium complexes did not show any signal according to the proton of the hydroxyl group. This is probably because of the formation of hydrogen bonding between the proton of the hydroxyl group of the ligand with acetate counter ion as in Scheme 4 [6]. Thus, the disappearance of the hydroxyl group protons might be because of the rapid fluctuation frequency of the hydrogen bonding between the oxygen atom of the ligand and the oxygen atom of the two acetate counter ions. The ^1^H NMR spectrum of the platinum complex (Fig. 5) displayed singlet signals at 12.89 ppm and 10.00 ppm referring to the protons of hydroxyl groups, which vanished with the addition of D_2_O. The signal at 12.89 ppm showed a noticeable shift from the ligand indicating the coordination of the aromatic OH to the metal center without proton displacement [5]. Furthermore, a singlet signal at 9.51 ppm appeared in the spectrum due to the proton of the azomethine group with a notable shift from the ligand. Multiplets from 7.25 ppm to 8.65 ppm were integrated for the aromatic protons. A singlet signal was shown in the ^1^H NMR spectrum at 3.95 ppm pointing out two protons of the methylene group.

**Fig. 6:**
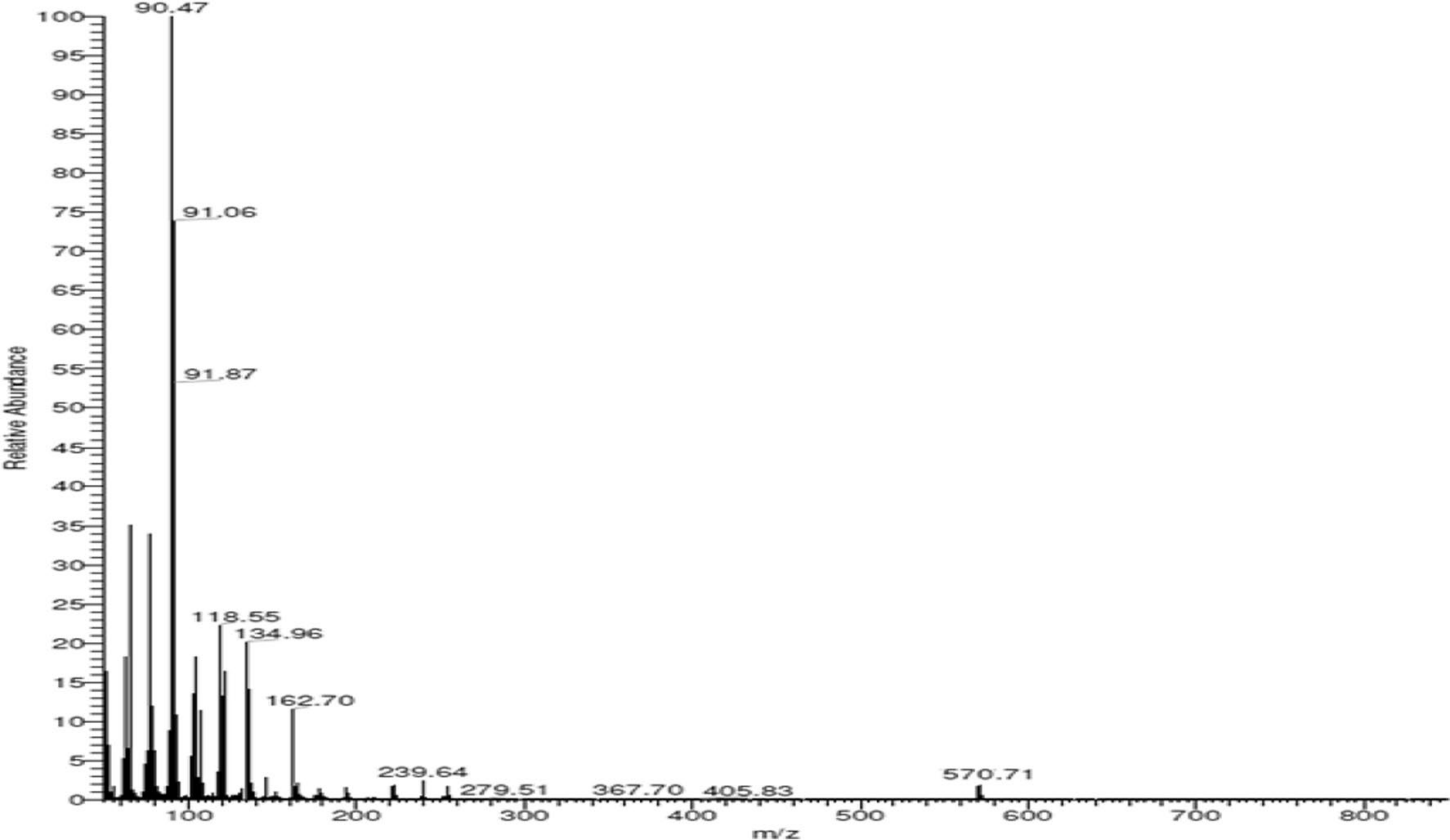
Mass spectrum of the platinum complex

**Fig. 7:**
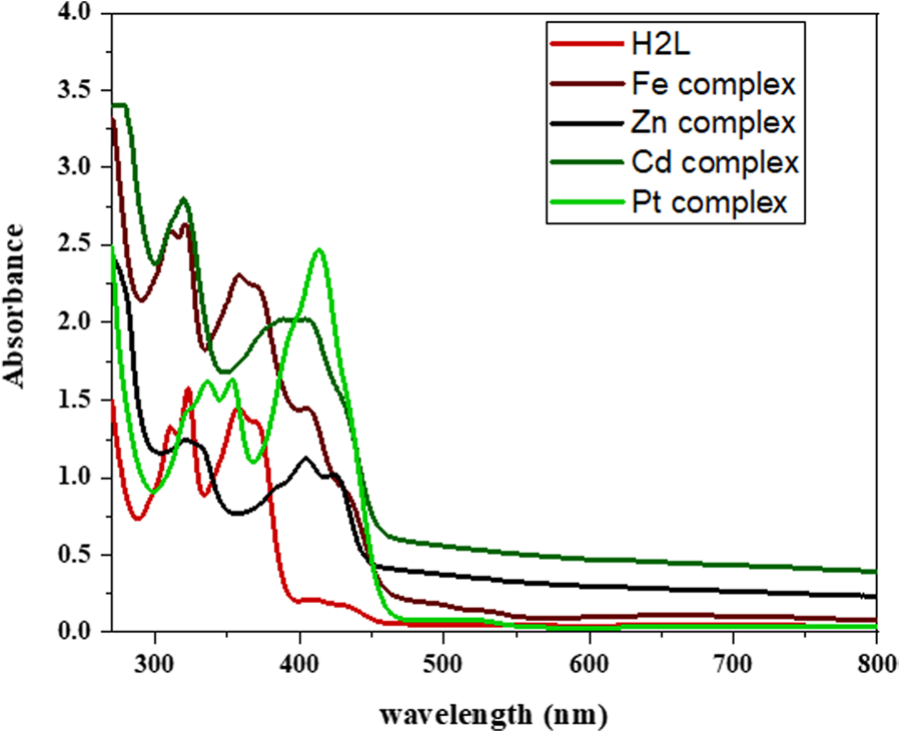
UV–Vis spectrum of the ligand and its complexes

The magnetic measurements of the iron complex revealed the paramagnetic character which reflected the weak field of the ligand. The effective magnetic moment (µ_eff_) of the iron complex was calculated to be 4.75 B.M., which is close to the spin-only moment of four unpaired electrons in the high spin *d*^*6*^ electronic configuration. EPR of the iron (II) complex has been carried out, but it has a broad spectrum because Fe(II) is EPR inactive (S = 2 in high spin case). This finding confirmed the + 2-oxidation state of the iron complex in a high spin field (S = 2, four unpaired electrons). Using electron paramagnetic resonance (EPR) spectroscopy, the electronic states of metal centers with half-integer spin states will be active. Nonetheless, under typical EPR settings, a substantial zero-field splitting (ZFS) frequently makes paramagnetic metal centers with integer spin EPR-silent. Table 1 shows the molar conductivity of DMF solutions of the complexes (10^−3^ M) at 25 °C. The conductance of the Zn and Cd complexes was 22 and 21 Ω^−1^mol^−1^cm^2^, indicating that these compounds have weak electrolytic characteristics. This could be because of the weak acetate counter ion. The iron and platinum complexes, on the other hand, had molar conductance of = 4.8 and 12.8 Ω^−1^mol^−1^cm^2^, showing that they were non-electrolytic [5]**.** One useful method for verifying the complexes' suggested formula is mass spectrometry. The ESI mass spectrum of the iron complex is represented in Fig. S6. It showed a parent peak at m/z 662.12 (calc. 662.51 amu) corresponding to [M]^+^. ESI–MS spectrum of zinc complex (Fig. S7) displayed a parent peak at m/z 791.96 amu according to [M-(CH3COO)]^+^. ESI–MS spectrum of cadmium complex (Fig. S8) displayed a molecular ion peak at m/z 831 attributed to [M-2(CH_3_COO)]^+^ ion. ESI–MS spectrum of platinum complex (Fig. 6) displayed a molecular ion peak at m/z 570.71 attributed to [M-2.5H_2_O]^+^ ion. The electronic absorption spectra of H_2_L ligand and its Fe(II), Zn(II), Cd(II), Pt(II) complexes are recorded in the wavelength range of 250–800 nm at 298 K and shown in Fig. 7. The ligand showed four absorption bands at 310, 322, 371, and 417 nm, these bands can be assigned to the π → π* transition of the benzene and naphthyl rings of the Schiff base ligand and n → π* of the imine and hydroxyl groups. Interestingly, these bands appeared at longer wavelengths due to the conjugation of the naphthyl group. Upon complexation, there is a shift in intensity and wavelength, indicating the formation of metal complexes. This is further supported by the appearance of bands at *λ* max = 518–528 nm, corresponding to charge transfer from the ligand to the metal ions. These charge transfers may occur between the p-orbitals of the imine ligand and the d-orbitals of metal ions. The electronic spectra of the iron complex showed a very low-intensity band that appeared at 670 nm; this band could be mostly ascribed to the d → d transition.

### Thermogravimetric studies

Thermal studies of the reported complexes were carried out using thermogravimetry (TG). The TG data of the reported complexes are depicted in (Table S1). The TG plots of [Zn_2_(HL)_2_](CH_3_COO)_2_ and [Cd_2_(HL)_2_](CH_3_COO)_2_ displayed the same decay pattern. The plots exhibited three resolved decomposition steps in the temperature range of 196–533 °C. The first decomposition occurred in the temperature range of 196–294 °C, with a net weight loss corresponding to the elimination of acetate moieties. On the other hand, the second and third decomposition steps of the two complexes were due to the loss of the organic content to leave the metallic oxide as a residue (2ZnO; found 19.147%; calc. 19.071%, 2CdO; found 27.051%; calc. 27.045%) [6]. The thermogravimetric curve of [Pt(H_2_L)Cl_2_] complex was found to be thermally decomposed through three well-defined decomposition steps within the temperature range of 31–495 °C. The first step occurred in the temperature range 31–212 °C with a net weight loss of 8.26% corresponding to the loss of 2.5 H_2_O moiety from the crystal lattice (calc. 7.91%). Moreover, the whole organic content was removed in the second and third degradation processes within the temperature range of 211–495 ⁰C to leave a PtO_2_ residue (found, 24.703%; calc. 24.96%).

The TG curve of the [Fe(HL)_2_] complex was found to be thermally decomposed in one decomposition step in the temperature range of 50–657 °C according to the elimination of the organic moiety to leave the metal iron as a final residue [5].

### Cytotoxicity activity

**Fig. 8:**
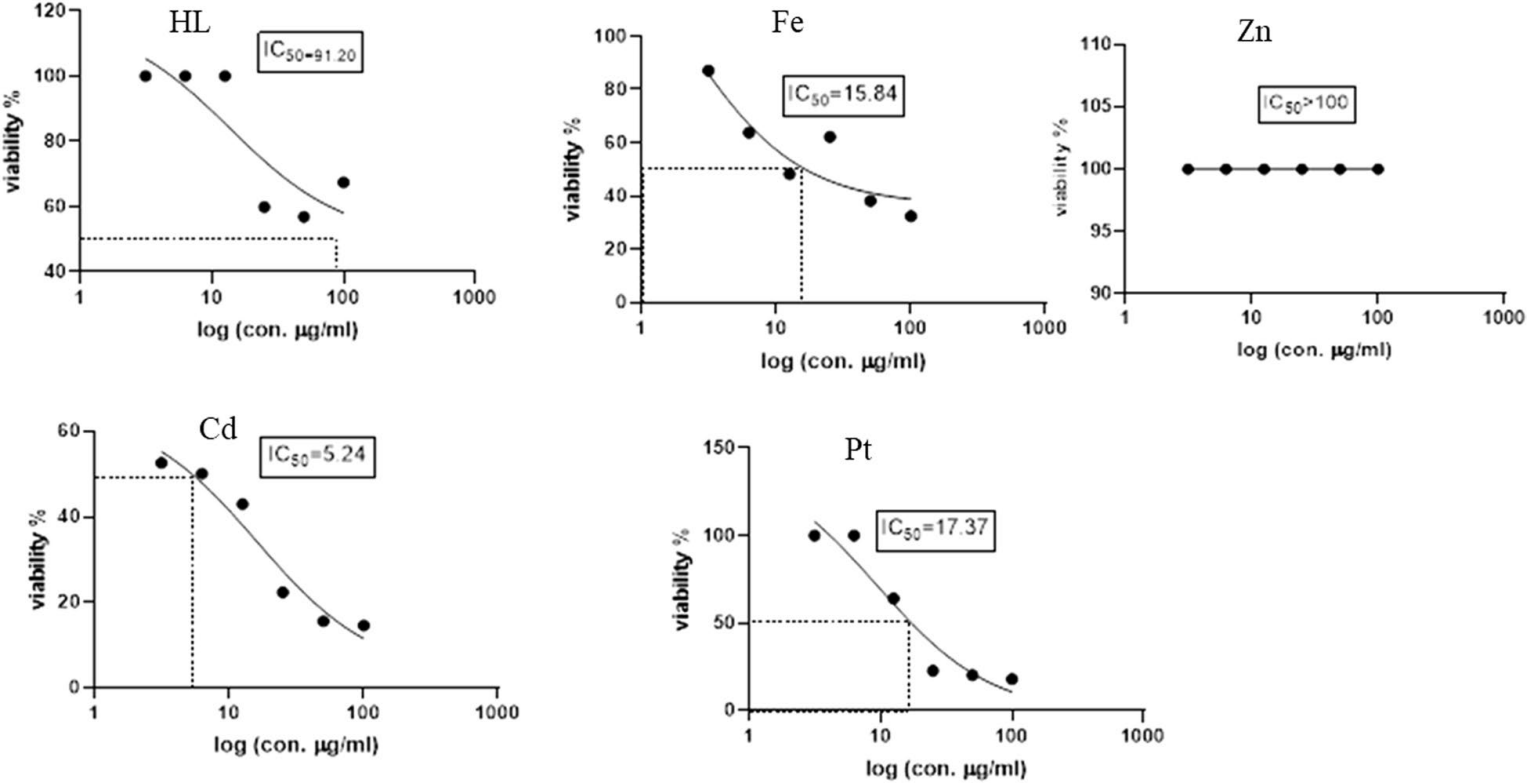
Dose–response curve and IC_50_ of Schiff-base (H_2_L) and its complexes on HSF normal human skin fibroblast

**Fig. 9:**
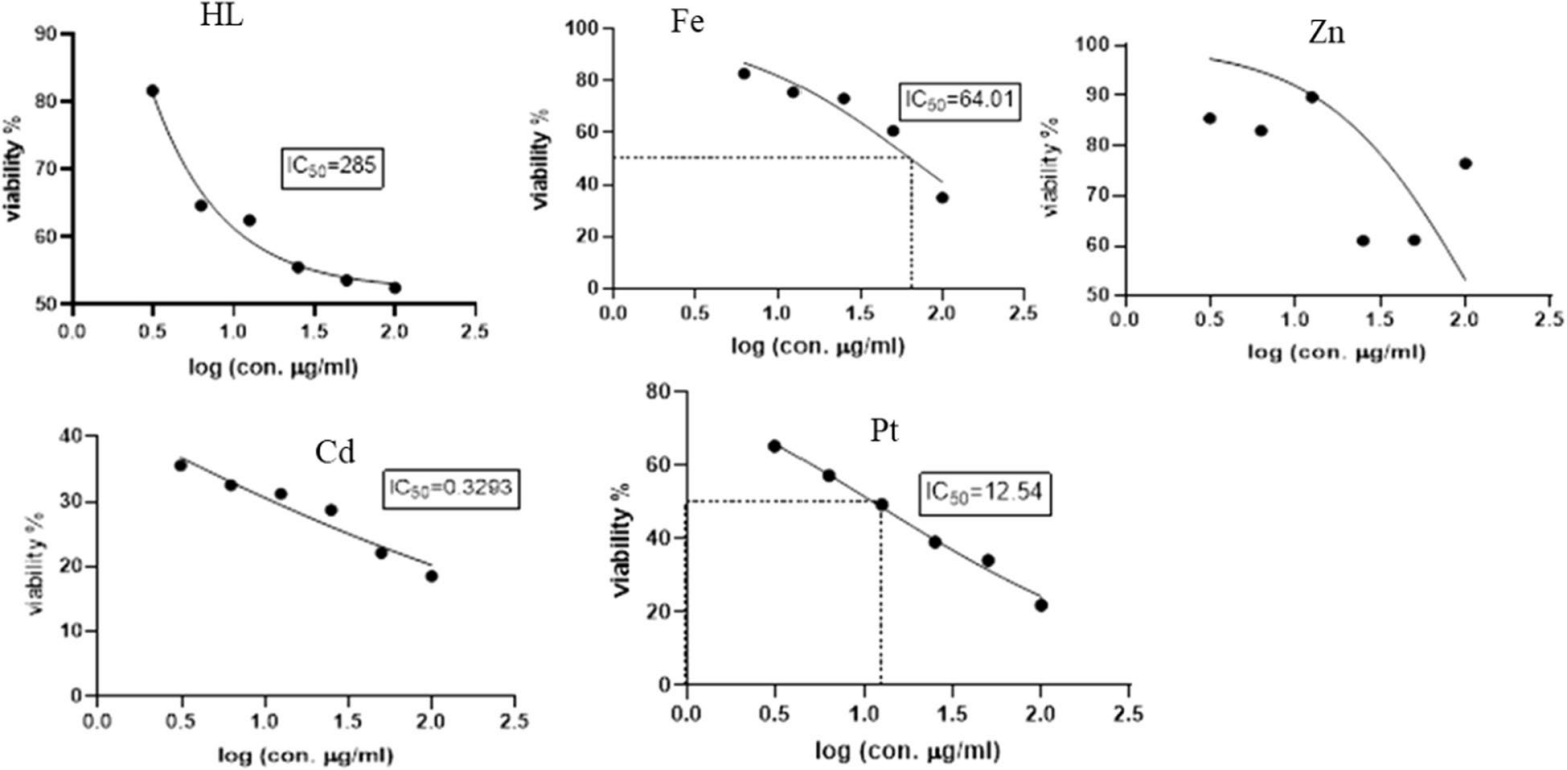
Dose–response curve and IC_50_ of Schiff-base (H_2_L) and its complexes on HCT119 (Human Colorectal cancer cell line)

**Fig. 10:**
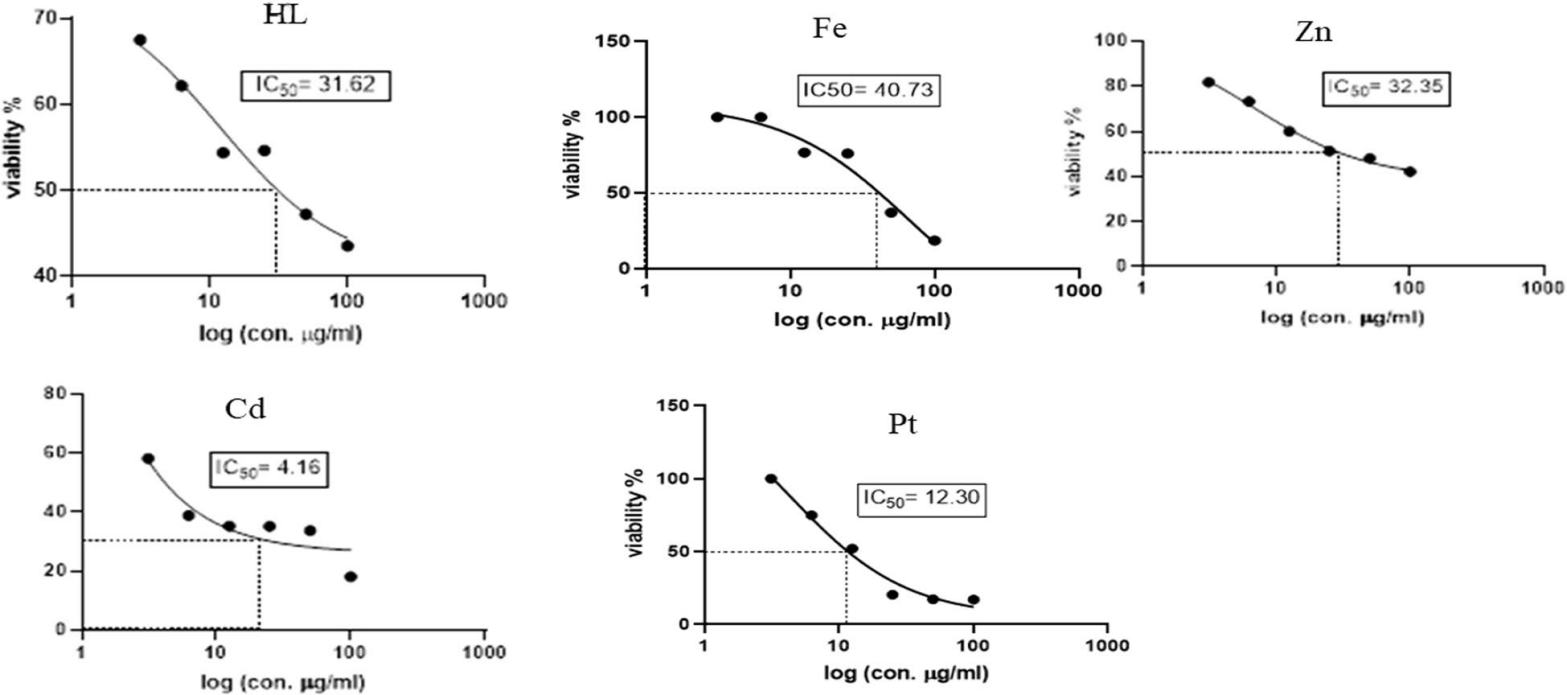
Dose–response curve and IC_50_ of Schiff-base (H_2_L) and its complexes on HepG2 (Human Liver cancer cell line)

**Fig. 11:**
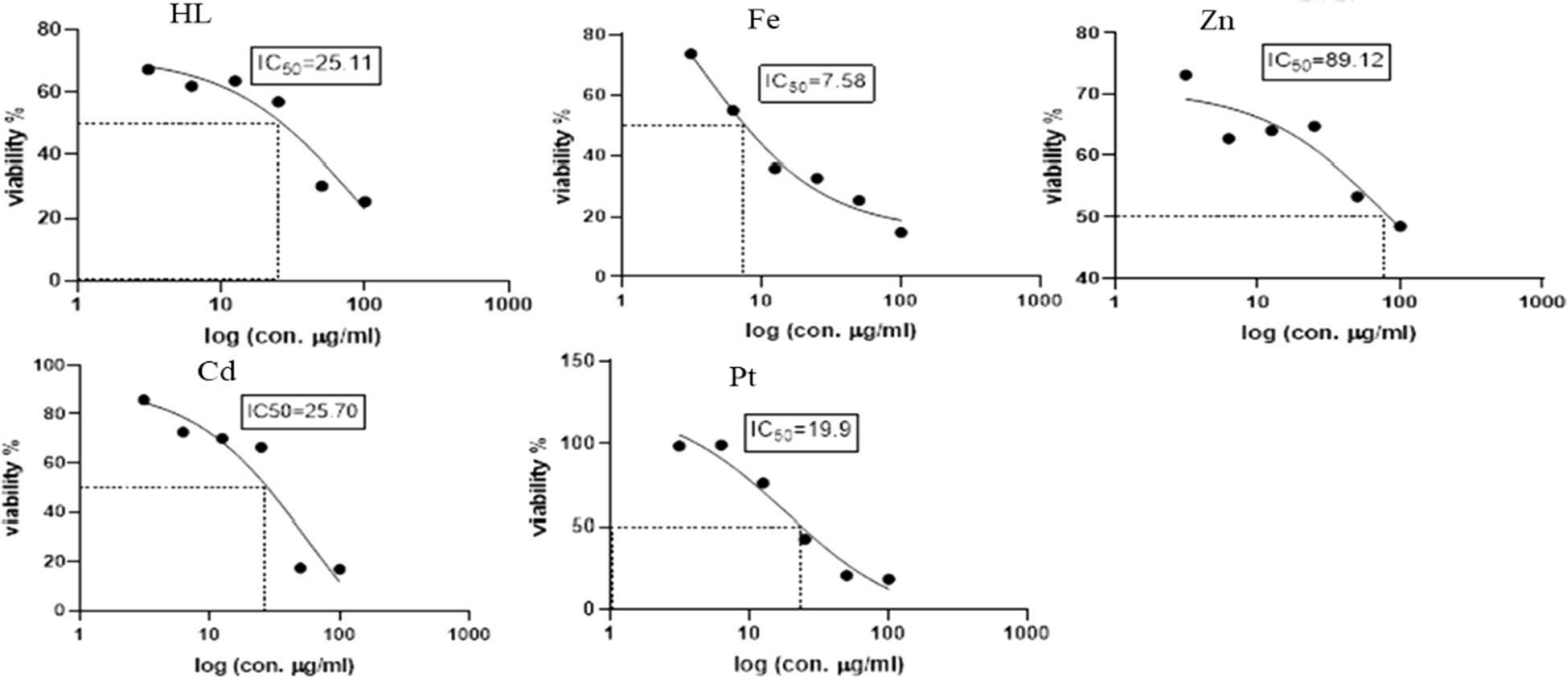
Dose–response curve and IC_50_ of Schiff-base (H_2_L) and its complexes on MDA (Human Breast cancer cell line)

**Fig. 12:**
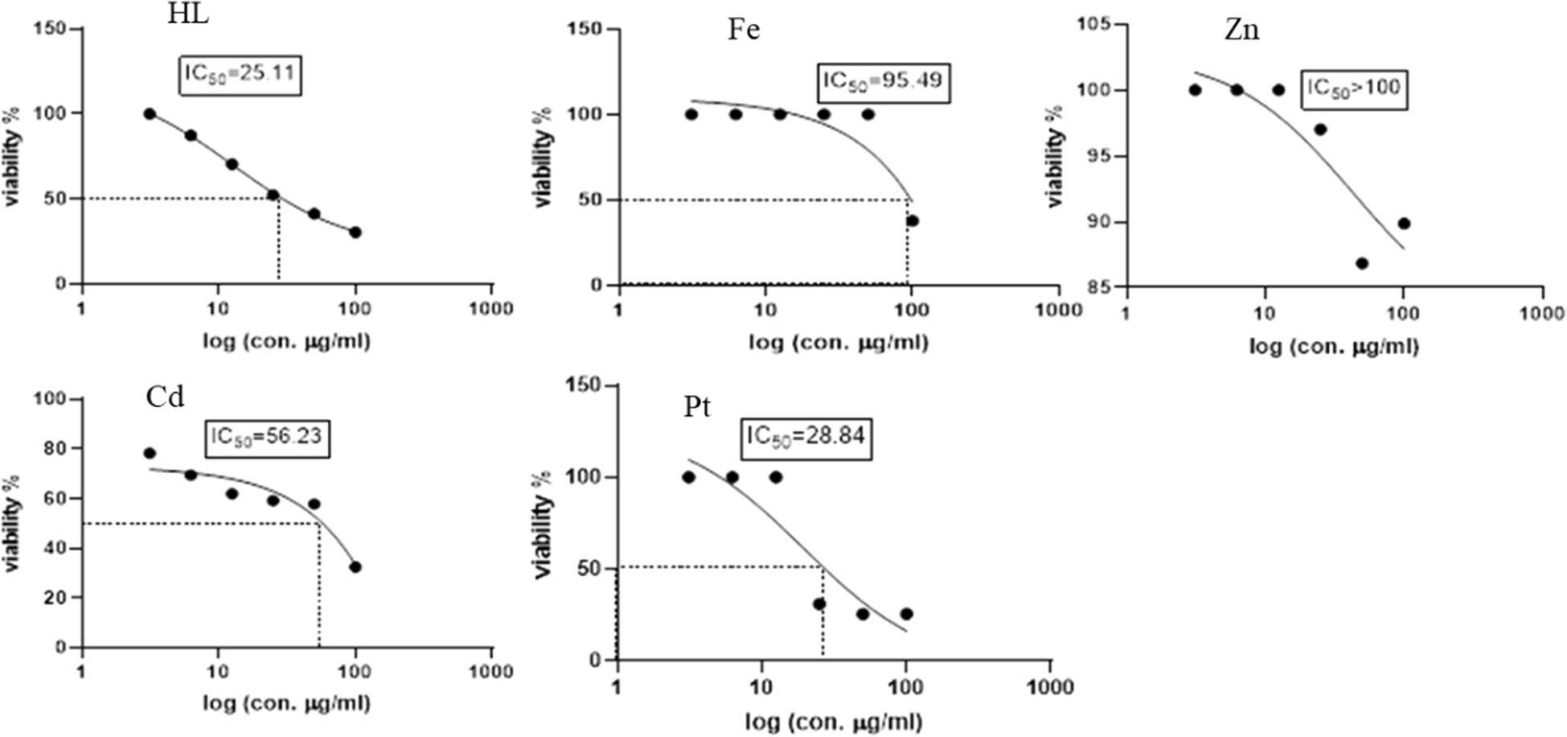
Dose–response curve and IC_50_ of Schiff-base (H_2_L) and its complexes on A549 (Human Lung cancer cell line)

To evaluate the potential usefulness of the Schiff base ligand (H_2_L) and the reported complexes (Fe, Zn, Cd, Pt) as antitumor agents, five human cell lines (normal Cells; HSF, human colorectal cancer cell line; HCT119, liver carcinoma cell line; HEPG2, human triple-negative breast cancer; MDA, human lung cancer cell line; A549) were treated by the compounds and compared with cis-platin activity as a standard. The IC_50_ values (concentration that produces 50% inhibition of cell growth) and selectivity index of the H_2_L ligand, the metal complexes, and cis-platin were tabulated in Table 4. Figures 8, 9, 10, 11 and 12 showed the dose–response curve and IC_50_ of the compounds against different cell lines.

**Table 4:** The IC_50_ values and selectivity index of the H_2_L ligand, the metal complexes, and the cis-platin

Compounds		HSF	HCT119	HEPG2	MDA	A549
H_2_L	*IC50*	91.2	285	31.62	25.11	25.11
	*SI*	1.00	0.32	2.88	3.63	3.63
Cd complex	*IC50*	**5.24**	**0.329**	**4.16**	25.7	56.23
	*SI*	1.00	15.93	1.26	0.20	0.09
Zn complex	*IC50*	100	100	32.35	89.12	100
	*SI*	1.00	1.00	3.09	1.12	1.00
Pt complex	*IC50*	17.37	12.54	12.3	19.9	28.84
	*SI*	1.00	1.39	1.41	0.87	0.60
Fe complex	*IC50*	15.84	64.01	40.73	7.58	95.49
	*SI*	1.00	0.25	0.39	2.09	0.17
Cisplatin	*IC50*	*11.07*	*2.25*	*3.42*	*3.21*	*2.73*
	*SI*	*1.00*	*4.92*	*3.24*	*3.45*	*4.05*

From the obtained data, one can emphasize that among the four tested metal complexes, cadmium complex showed the best sensitivity and selectivity against the human colorectal cancer cell line (HCT119) and human liver cancer cell line (HEPG2) with (IC_50_ = 0.329 µg/ml, selectivity index = 15.93) and (IC_50_ = 4.16 µg/ml and selectivity index = 1.26) respectively. Regarding the human triple-negative breast cancer (MDA), iron complex had the best effect with IC_50_ = 7.58 µg/ml and selectivity index = 2.09, However, it showed the least promising result against human liver cancer cell line (HEPG2) “IC_50_ = 40.73 and selectivity index = 0.39” and human lung cancer cell line (A549) “IC_50_ = 95.49 and selectivity index = 0.17”. Concerning the human lung cancer cell line (A549), the Schiff base ligand H_2_L showed the best effect with IC_50_ = 25.11 µg/ml and selectivity index = 3.63. Yet, it had the least impact on the human colorectal cancer cell line (HCT-119) with IC_50_ = 285 µg/ml and selectivity index = 0.32. It’s also worth mentioning that the Zn complex was the least promising drug among the four tested metal complexes against human triple-negative breast cancer (MDA) with IC_50_ = 89.12 µg/ml and selectivity index = 1.12. On the other hand, the present data showed zinc complex had less Cytotoxicity on HCT-119 and A549 when compared to those reported by [42]. It’s worth mentioning that cadmium complex is the most recommended drug as it has the least IC_50_, less than the positive control “Cisplatin,” and the highest selectivity index. Regarding platinum complex, it can be deduced from the previous data, that it also had promising anti-cancer effects as it came in second place regarding the cytotoxicity against HCT119, MDA, and A549 with IC_50_ = 12.54, 19.9, 28.84, and SI = 1.39, 0.87, 0.6, respectively.

We can also conclude that the cytotoxic effect can be correlated to the various elements used, the stereochemistry of the Schiff base, and the fact that different cell lines have different cell surface receptors.

### Docking studies

The binding free energy of the ligand and complexes with the receptor of Methionine adenosyltransferase (MAT) in liver cancer (PDB ID: 5A19) are found to be − 6.4, − 23.8, − 16.3, − 30.0 and − 33.1 kCal/mol for the Ligand, [Pt(H_2_L)Cl_2_], [Fe(HL)_2_], [Zn_2_(HL)_2_]^2+^ and [Cd_2_(HL)_2_]^2+^; respectively, Table S2. The more negative the binding energy the stronger interaction.

$$ \left[ {{\text{Cd}}_{{2}} \left( {{\text{HL}}} \right)_{{2}} } \right]^{{{2} + }} > \left[ {{\text{Zn}}_{{2}} \left( {{\text{HL}}} \right)_{{2}} } \right]^{{{2} + }} > \left[ {{\text{Pt}}\left( {{\text{H}}_{{2}} {\text{L}}} \right){\text{Cl}}_{{2}} } \right] > \left[ {{\text{Fe}}\left( {{\text{HL}}} \right)_{{2}} } \right] > {\text{H}}_{{2}} {\text{L}}. $$

**Fig. 13:**
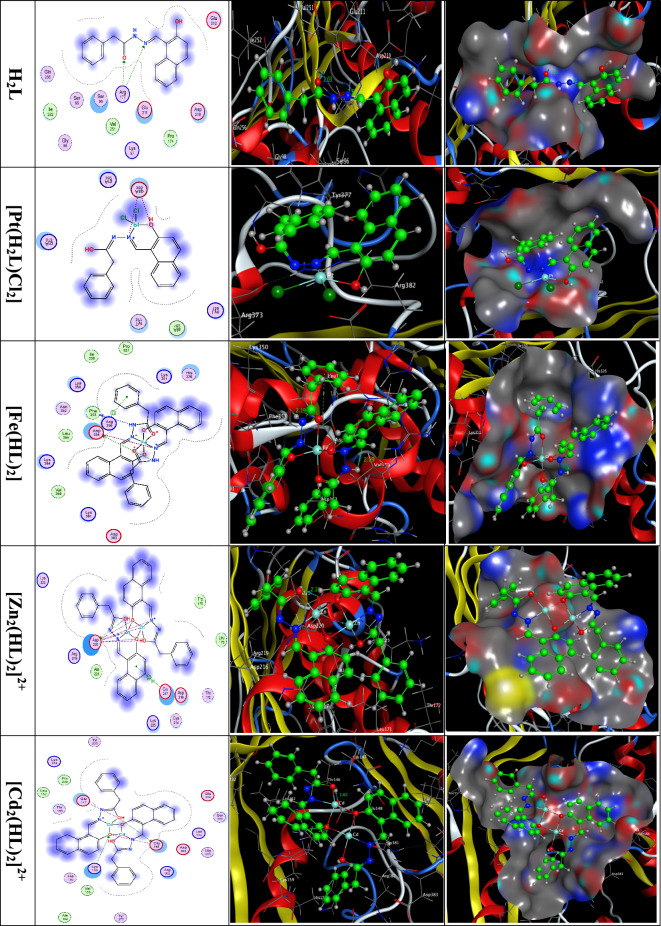
2D and 3D plots of the interaction between the Ligand, [Pt(H_2_L)Cl_2_], [Fe(HL)_2_], [Zn_2_(HL)_2_]^2+^ and [Cd_2_(HL)_2_]^2+^ with the active site of the receptor of liver cancer protein (PDB ID: 5A19). Hydrophobic interactions with amino acid residues are shown with dotted curves

The 2D and 3D plots of the interaction of the Ligand, [[Pt(H_2_L)Cl_2_], [Fe(HL)_2_], [Zn_2_(HL)_2_]^2+^ and [Cd_2_(HL)_2_]^2+^ with the active sites of the receptor of liver cancer protein (PDB ID: 5A19) are shown in Fig. 13. The docking active sites of the Cd-complex are mainly located on the Glu and Lys amino acids and those of the Zn-complex are mainly Asp amino acids. Also, the docking active sites of the Pt-complex are located on the Arg and Asp amino acids and those of the Fe-complex are mainly Asp and Phe amino acids. The interactions involve strong hydrogen bonds (Table S2 and Fig. 13).

### Molecular DFT studies

#### Molecular DFT studies of ligand (H_2_L)

**Fig. 14:**
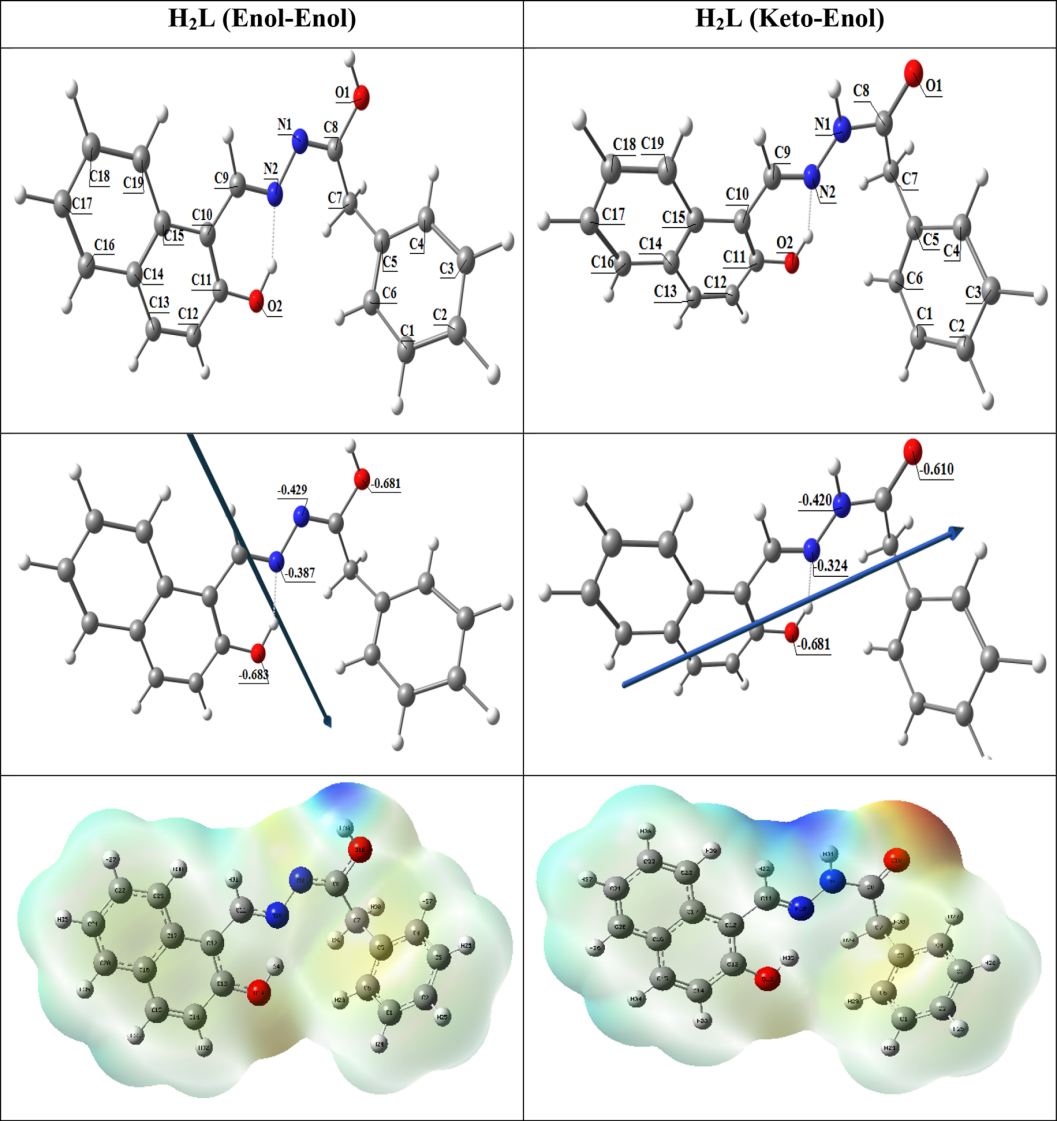
The optimized structure of the ligand, the vector of the dipole moment, the natural charges on atoms, and the molecular electrostatic potential (MEP) surface by density function B3LYP/6-311g^++^(d, p)

Figure 14 shows the optimized structures of the ligand as the lowest energy configurations. The Keto-Enol form is slightly more stable than the Enol-Enol form by − 0.011 Hartree, or − 0.2993 eV. The natural charges obtained from Natural Bond Orbital Analysis (NBO) show that the more negative active sites are O2 (− 0.681) > O1 (− 0.610) > N1 (− 0.420) > N2 (− 0.324) for Keto-Enol form and O2 (− 0.683) > O1 (− 0.681) > N1 (− 0.429) > N2 (− 0.387) for Enol-Enol form. So, atoms O1, O2, and N2 may coordinate with metal ions. The O2-H forms H-bonds with N2 in both isomers thus the H atom is close to N2 and O2.

##### Molecular DFT studies of complexes

Figure S9 shows the optimized lowest energies structures of the complexes [Pt(H_2_L)Cl_2_], [Fe(HL)_2_], [Zn_2_(HL)_2_]^2+^ and [Cd_2_(HL)_2_]^2+^; respectively. The metal atoms form six-coordinated in distorted octahedral geometries for all complexes except for [Pt(H_2_L)Cl_2_] is square planar. The atoms (N2, O1, Cl2, and Cl1) in [Pt(H_2_L)Cl_2_] and (N2, O2, O4, and O3) in [Fe(HL)_2_] are almost in one plane deviated by − 0.117° and − 7.901°; respectively. The atoms (N1, O1, O2 and O4) in [Zn_2_(HL)_2_]^2+^ and [Cd_2_(HL)_2_]^2+^ are deviated by 7.801° and − 4.113°; respectively. The atoms (N3, O3, O1 and O2) in [Zn_2_(HL)_2_]^2+^ and [Cd_2_(HL)_2_]^2+^ are deviated by 8.105° and 4.598°, respectively; Tables S3.

**Fig. 15:**
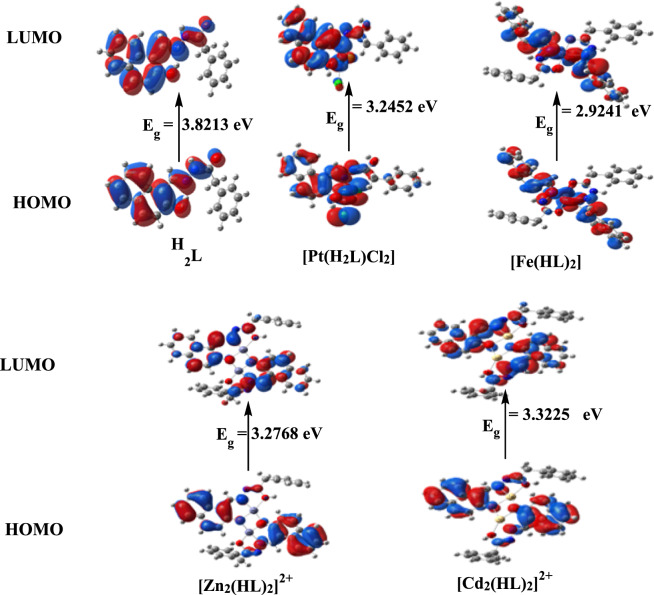
HOMO and LUMO charge density maps of Ligand, [Pt(H_2_L)Cl_2_], [Fe(HL)_2_], [Zn_2_(HL)_2_]^2+^ and [Cd_2_(HL)_2_]^2+^

The computed total energy, the highest occupied molecular orbital (HOMO) energies, the lowest unoccupied molecular orbital (LUMO) energies, and the dipole moment for the ligands and complexes were calculated, in Table 5. The more negative values of the total energy of the complexes than that of the free ligand indicated that the complexes are more stable than the free ligand and the energy gap (E_g_) = E_LUMO_—E_HOMO_ is smaller in case of complexes than that of ligand due to chelation of ligand to metal ions, Table 5. The lowering of E_g_ in complexes compared to that of the ligand explains the charge transfer interactions upon complex formation, Fig. 15.

**Table 5:** Calculated energies and properties of H_2_L(Keto-Enol), H_2_L(Enol-Enol), and complexes [Pt(H_2_L)Cl_2_], [Fe(HL)_2_], [Zn_2_(HL)_2_]^2+^ and [Cd_2_(HL)_2_]^2+^

Property	H_2_L(Keto-Enol)	H_2_L(Enol-Enol)	[Pt(H_2_L)Cl_2_]	[Fe(HL)_2_]	[Zn_2_(HL)_2_]^2+^	[Cd_2_(HL)_2_]^2+^
E (a.u.)	− 993.875	− 993.864	− 2033.325	− 2109.415	− 2116.791	− 2081.677
HOMO (eV)	− 6.0045	− 5.8649	− 6.4564	− 5.1152	− 10.9926	− 10.8199
LUMO (eV)	− 2.1832	− 2.0741	− 3.2112	− 2.1911	− 7.7158	− 7.4974
E_g_ (eV)	3.8213	3.7908	3.2452	2.9241	3.2768	3.3225
Dipole moment (Debye)	3.5389	1.0872	12.7423	2.0595	0.3000	0.4378
I = − E_HOMO_	6.0045	5.8649	6.4564	5.1152	10.9926	10.8199
A = − E_LUMO_	2.1832	2.0741	3.2112	2.1911	7.7158	7.4974
χ = (I + A)/2	4.0938	3.9695	4.8338	3.6531	9.3542	9.1586
η = (I − A)/2	1.9106	1.8954	1.6226	1.4620	1.6384	1.66125
S = 1/2η	0.2617	0.2638	0.3081	0.3420	0.3052	0.3010
μ = − χ	− 4.09385	− 3.9695	− 4.8338	− 3.6531	− 9.3542	− 9.1586
ω = μ^2^/2η	4.3858	4.1566	7.2001	4.5640	26.7032	25.2463

**Reactivity studies**

Many reactivity descriptors such as ionization potential (I), electron affinity (A), Electronegativity (χ), chemical potential (μ), hardness (η), softness (S), and electrophilicity index (ω), all derived from the HOMO and LUMO energies, have been proposed for understanding various aspects of reactivity associated with chemical reactions, Table 5.

## Conclusion

In conclusion, this study successfully introduces a novel Schiff base, N'-((2-hydroxynaphthalen-1-yl)methylene)-2-phenylacetohydrazide (H_2_L), together with its metal complexes; [Fe(HL)_2_], [Zn_2_(HL)_2_](CH_3_COO)_2_, [Cd_2_(HL)_2_](CH_3_COO)_2_, and [Pt(H_2_L)Cl_2_]. These complexes were characterized using various spectroscopic techniques (IR, ^1^H NMR), molar conductivity, CHN analyses, magnetic measurements, and thermal analysis. The spectroscopic techniques proved the octahedral structure of Fe(II) complex, whereas, Pt(II) complex exhibited square planar geometry. The zinc and cadmium complexes existed in a dimeric structure where two metal ions were coordinated with two bridging oxygen atoms from two ligand molecules. This research delved into the study of the effect of different complexes on different cancerous cell lines. Five human cell lines (normal Cells; HSF, human colorectal cancer cell line; HCT119, liver carcinoma cell line; HEPG2, human triple-negative breast cancer; MDA, human lung cancer cell line; A549) were treated by the compounds and compared with cis-platin activity as a standard. Among the four tested metal complexes, cadmium complex showed the best sensitivity and selectivity against the human colorectal cancer cell line (HCT119) and human liver cancer cell line (HEPG2) with (IC_50_ = 0.329 µg/ml, selectivity index = 15.93) and (IC_50_ = 4.16 µg/ml and selectivity index = 1.26) respectively. Other complexes in this study exhibited comparable activity toward different cell lines used in this research. This result manifested these complexes are outstanding candidates for antitumor activity. The optimized molecular geometry of the complexes was computed by DFT-B3LYP/LANL2DZ level of theory. To examine how the ligand and its complexes interact with methionine adenosyl-transferases in liver cancer (PDB ID: 5A19), molecular docking was used. According to the docking investigation, the Cd(II) complex had the strongest affinity for the cancer cell target 5A19, with a high negative energy S-score value. Ultimately, the study's overall findings suggested that these complexes would be more successful in demonstrating anti-cancer properties.

## Supplementary Information

Supplementary Material 1.

## Data Availability

No datasets were generated or analysed during the current study.
