# Supplementary material for: Spectroscopic, docking, antiproliferative, and anticancer activity of novel metal derivatives of phenylacetohydrazide Schiff base on different human cancer cell lines
Source: BMC Chem. 2025 Mar 15;19(1):69. doi: 10.1186/s13065-025-01417-1 (PMC11909975; doi:10.1186/s13065-025-01417-1)
Supplement: Supplementary file 1 — Supplementary Material 1. [file 13065_2025_1417_MOESM1_ESM.docx]

**Spectroscopic, Docking, Antiproliferative, and Anticancer activity of novel metal derivatives of** **phenylacetohydrazide Schiff base on different human cancer cell lines**

Manal A. Afifi^1^, Anas A. Rasmy^2^, Emad M. Elzayat^2^, Samir M. El-Medani^1^, Mohamed R. Shehata^3^, Fatma M. Elantabli^1,*^

^1^Chemistry Department, Faculty of Science, El-Fayoum University, El-Fayoum, Egypt.

^2^Department of Biotechnology, Faculty of Sciences, Cairo University, Giza, Egypt.

^3^Chemistry Department, Faculty of Science, Cairo University, Giza, Egypt.

Corresponding author: Fatma M. Elantabli

e-mail: [fma01@fayoum.edu.eg](mailto:fma01@fayoum.edu.eg)

**Supplementary file**


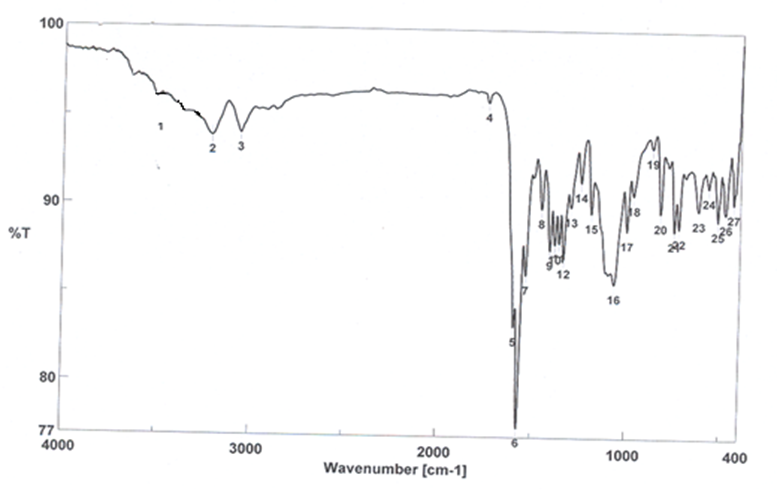


Fig. S1. IR spectrum of iron complex.


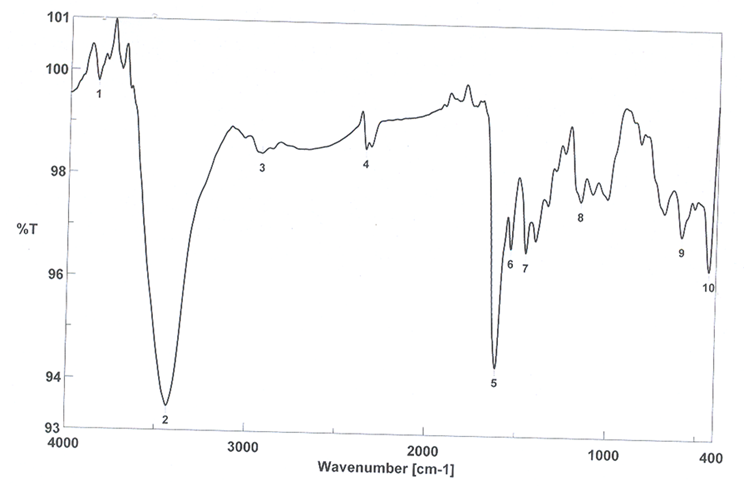


Fig. S2. IR spectrum of zinc complex.


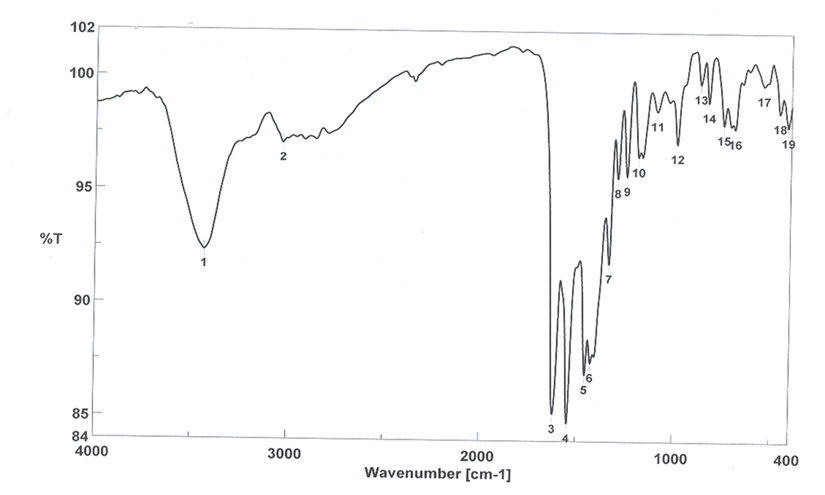


Fig. S3. IR spectrum of cadmium complex.


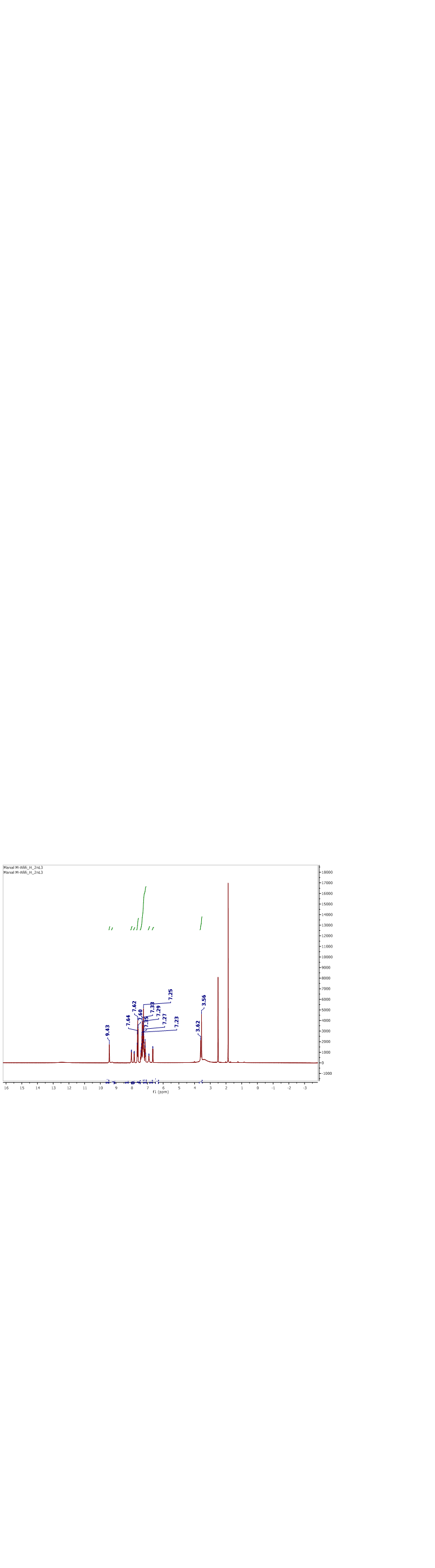


Fig. S4. ^1^HNMR spectrum of zinc complex.


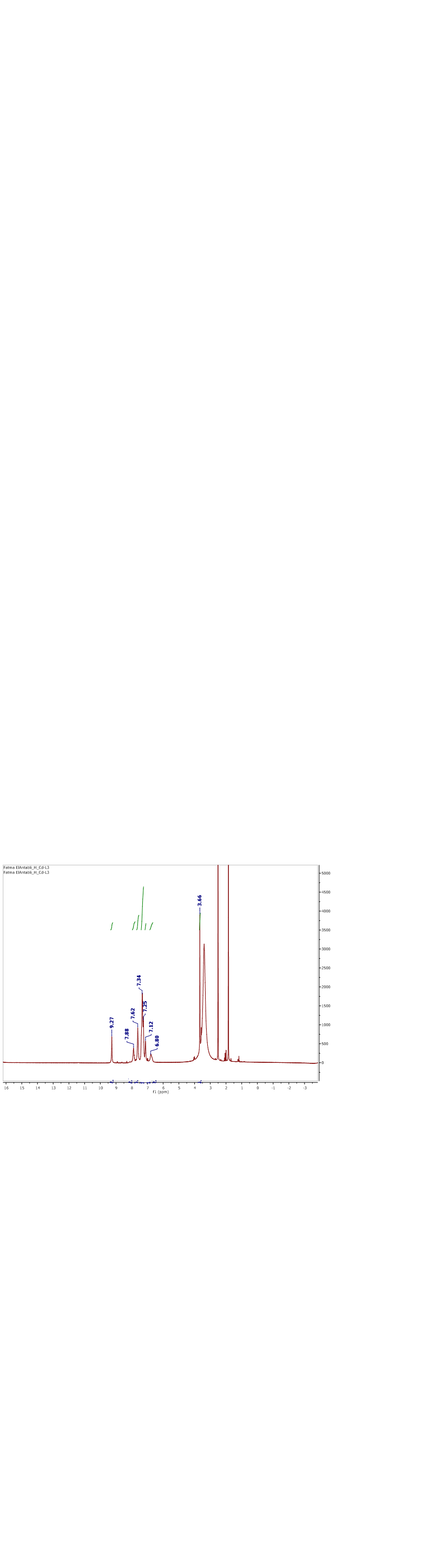


Fig. S5. ^1^HNMR spectrum of cadmium complex.


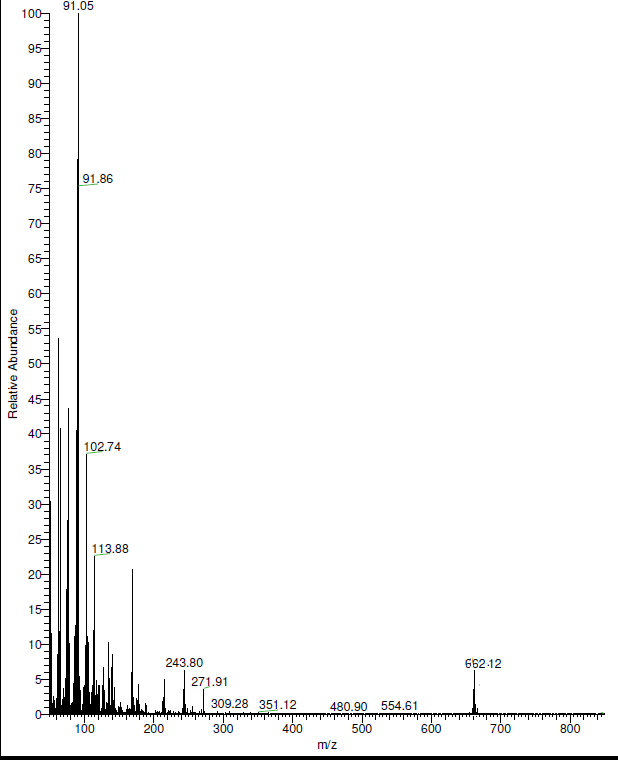


Fig. S6. Mass spectrum of iron complex.


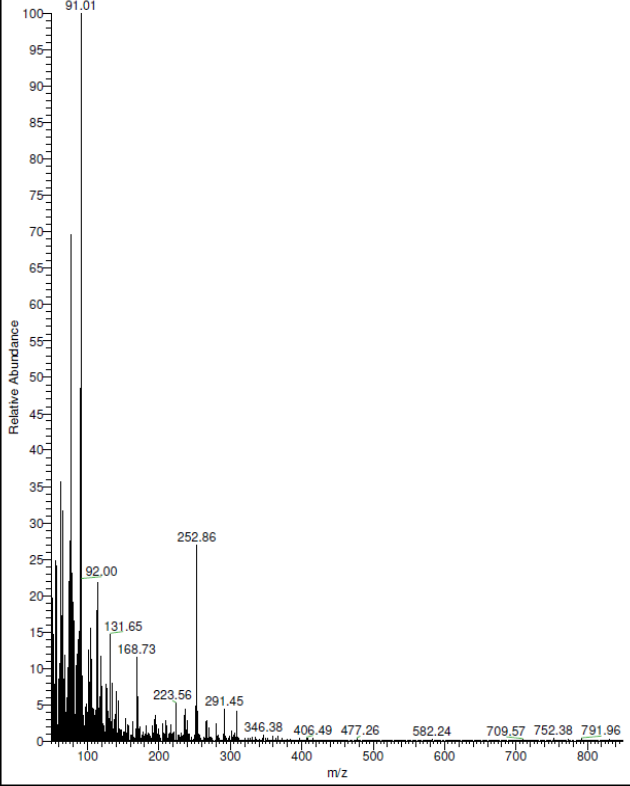


Fig. S7. Mass spectrum of zinc complex.


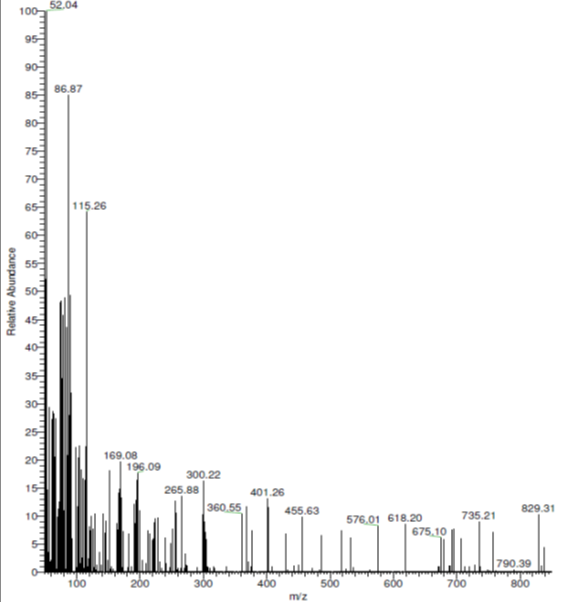


Fig. S8. Mass spectrum of cadmium complex.

| 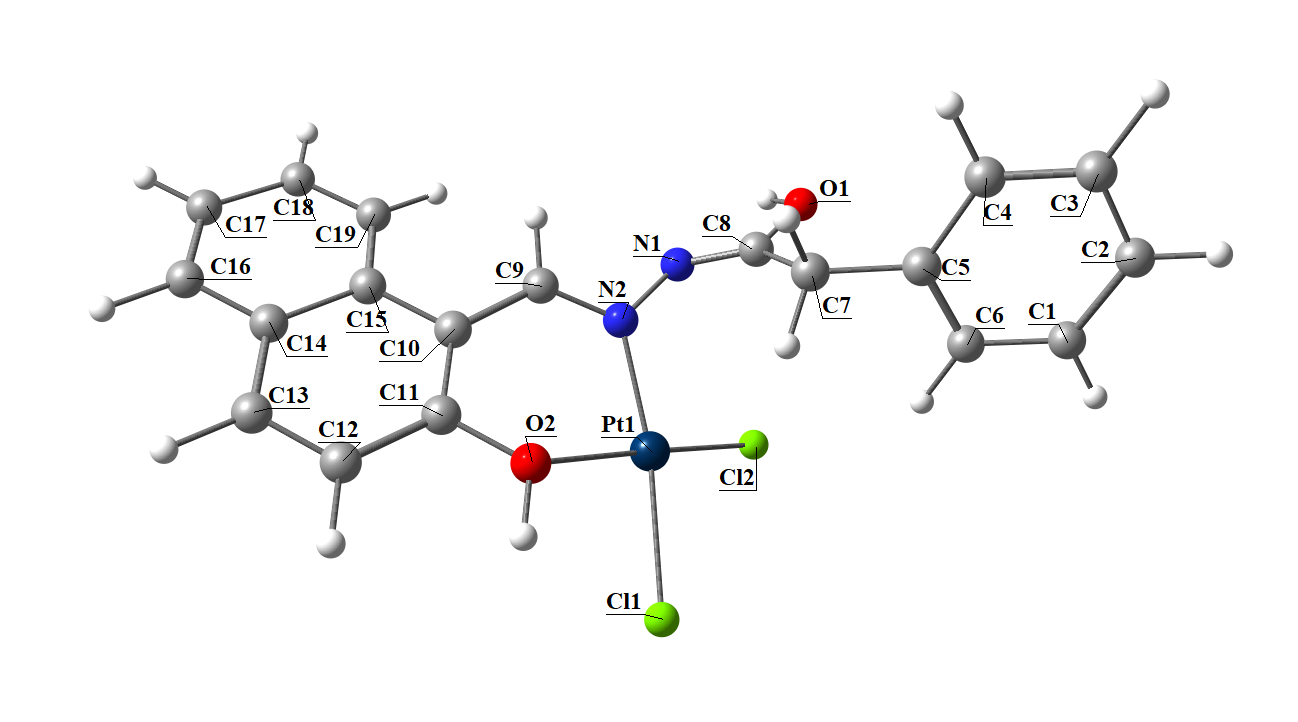 | 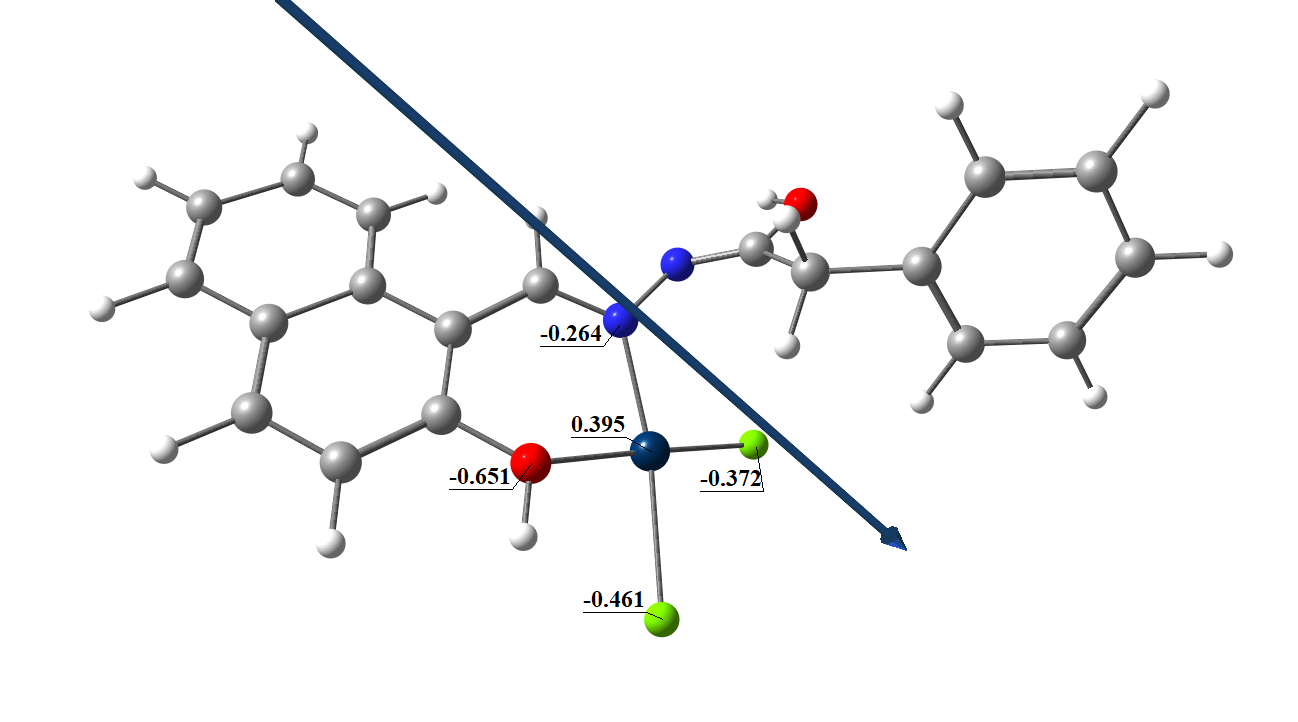 |
| --- | --- |
| 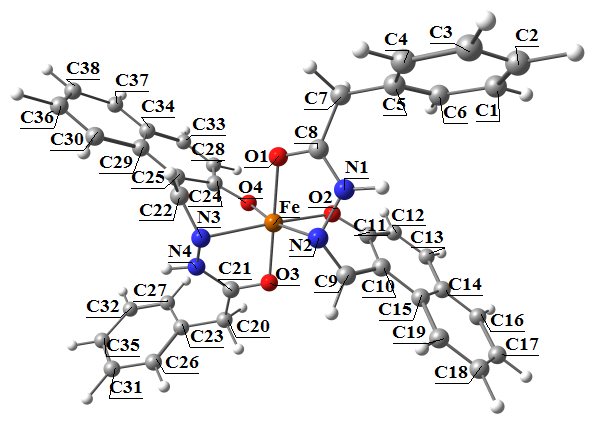 | 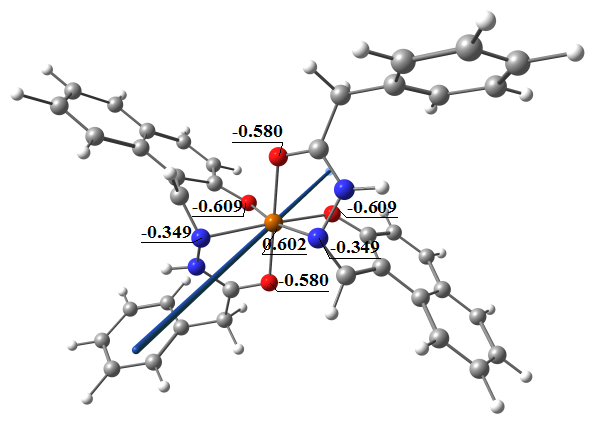 |
| 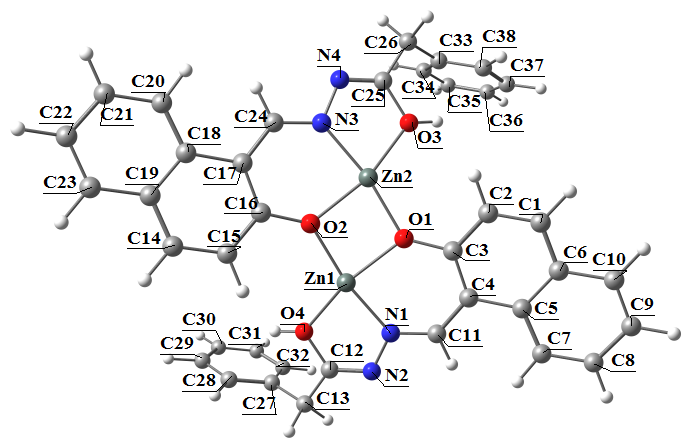 | 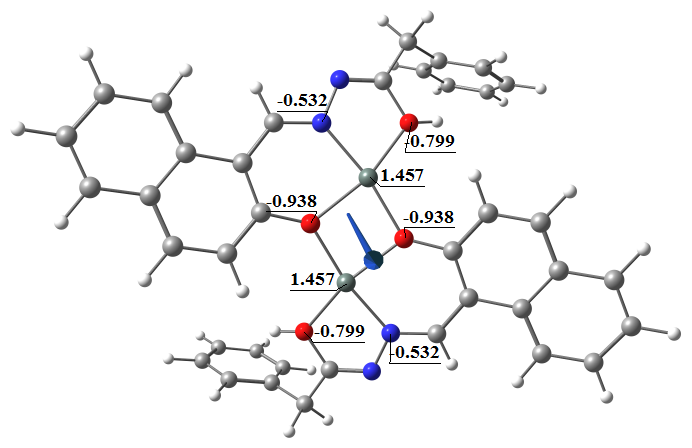 |
| 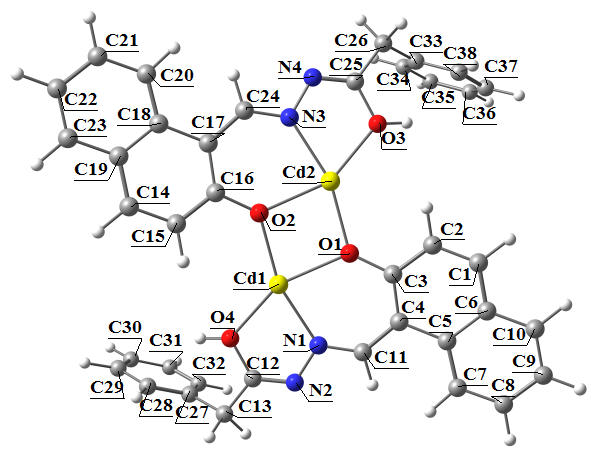 | 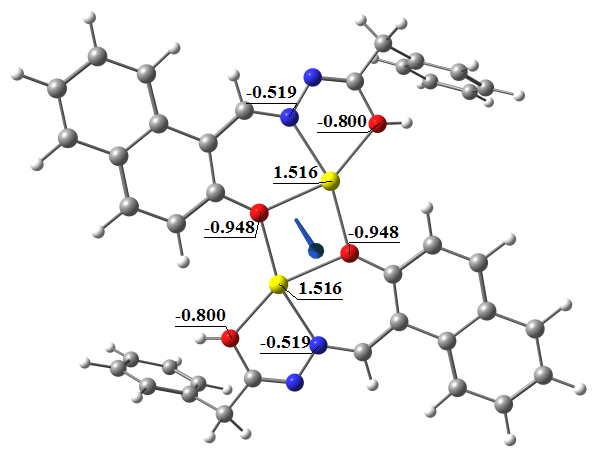 |

**Fig. S9.** The optimized structures, the vector of the dipole moment, and the natural charges on coordinated atoms of [Pt(H_2_L)Cl_2_], [Fe(HL)_2_], [Zn_2_(HL)_2_]^2+^ and [Cd_2_(HL)_2_]^2+^ complexes.

**Table S1.** The thermogravimetric data for the reported complexes.

| Molecular formula | M.M. | Decomposition temperature, ^o^C | % Weight loss | Eliminated species | % Solid residue,  Found (cal.) |
| --- | --- | --- | --- | --- | --- |
|  |  |  |  |  |  |
| Zn_2_C_42_H_36_N_4_O_8_ | 855.52 | 196-294  294-364  364-533 | 6.703%  18.765%  55.79% | 2CH_3_COO  C_8_H_18_NO_2_  C_32_H_15_N_3_O_2_ | 2ZnO  19.147 (19.071) |
| Cd_2_C_42_H_36_N_4_O_8_ | 949.58 | 179-304  304-427  427-582 | 12.9%  43.65%  16.125% | 2CH3COO  C_30_H_14_N_3_  C_8_H_16_ NO_2_ | 2CdO  27.051 (27.045) |
| PtC_19_H_21_N_2_O_4.5_Cl_2_ | 615.37 | 31-212  211-495 | 8.26%  60.89% | 2.5H_2_O  C_19_H_16_N2Cl2 | PtO_2_  31.08 (31.96) |
| FeC_38_H_30_N_4_O_4_ | 662.51 | 50-657 | 92.40 | C_38_H_30_N_4_O_4_ | Fe  7.6  (7.8%) |

**Table S2.** The Docking interaction data calculations of Ligand, [Pt(H_2_L)*Cl*_2_], [Fe(HL)_2_], [Zn_2_(HL)_2_]^2+^ and [Cd_2_(HL)_2_]^2+^ with the active sites of the receptor of liver cancer protein (PDB ID: 5A19).

|  | Receptor | Interaction | Distance(Å)* | E (kCal/mol) |
| --- | --- | --- | --- | --- |
| H_2_L | | | | |
| O 18 | NH2 ARG 177 | H-acceptor | 2.90 (2.03) | -4.7 |
| N 10 | NH1 ARG 177 | H-acceptor | 3.54 (2.55) | -1.7 |
| [Pt(H_2_L)*Cl*_2_] | | | | |
| O 19 | O ARG 168 | H-donor | 3.12 (2.48) | -10.2 |
| PT 39 | OD2 ASP 93 | Metal | 2.48 | -4.7 |
| O 20 | OD2 ASP 93 | Ionic | 2.94 | -8.9 |
| [Fe_2_(HL)_2_] | | | | |
| N 9 | OD2 ASP 354 | H-donor | 2.88 (2.19) | -3.5 |
| N 47 | O PHE 353 | H-donor | 3.22 (2.19) | -3.7 |
| N 9 | OD2 ASP 354 | Ionic | 2.88 | -5.3 |
| N 11 | OD2 ASP 354 | Ionic | 3.32 | -2.7 |
| 6-ring | CA ASP 354 | pi-H | 3.92 | -1.1 |
| [Zn_2_(HL)_2_]^2+^ | | | | |
| O 56 | OD1 ASP 220 | H-donor | 2.93 (2.10) | -6.8 |
| Zn 43 | OD2 ASP 220 | Metal | 2.27 | -2.1 |
| O 14 | OD1 ASP 220 | Ionic | 3.77 | -1.0 |
| O 14 | OD2 ASP 220 | Ionic | 2.68 | -4.0 |
| O 34 | OD1 ASP 220 | Ionic | 2.88 | -2.3 |
| N 37 | OD2 ASP 220 | Ionic | 3.26 | -1.9 |
| O 41 | OD2 ASP 220 | Ionic | 2.66 | -7.2 |
| O 56 | OD1 ASP 220 | Ionic | 2.93 | -5.0 |
| 6-ring | CG GLU 217 | pi-H | 3.98 | -0.7 |
| [Cd_2_(HL)_2_]^2+^ | | | | |
| O 45 | OE1 GLU 148 | H-donor | 2.64 (1.62) | -19.5 |
| O 38 | NZ LYS 159 | H-acceptor | 3.09 (2.19) | -1.8 |
| O 38 | OE2 GLU 148 | Ionic | 3.91 | -0.7 |
| O 45 | OE1 GLU 147 | Ionic | 3.28 | -2.9 |
| O 45 | OE1 GLU 148 | Ionic | 2.64 | -7.4 |
| O 45 | OE2 GLU 148 | Ionic | 3.85 | -0.8 |

*The lengths of H-bonds are in brackets.

**Table S3.** Important optimized bond lengths (Å) and bond angles (°) of the complexes [Pt(H_2_L)Cl_2_], [Fe(HL)_2_], [Zn_2_(HL)_2_]^2+^ and [Cd_2_(HL)_2_]^2+^.

| Bond lengths | [Pt(H_2_L)Cl_2_] | [Fe(HL)_2_] | [Zn_2_(HL)_2_]^2+^ | [Cd_2_(HL)_2_]^2+^ |
| --- | --- | --- | --- | --- |
| M1-N1 | - | - | 2.000 | 1.787 |
| M2-N3 | - | - | 2.000 |  |
| M1-N2 | 2.033 | 2.129 | - | - |
| M1-N3 | - | 2.129 | - | - |
| M1-O1 | - | 2.002 | 2.053 |  |
| M2-O1 | - |  | 1.969 |  |
| M1-O2 | 2.144 | 2.016 | 1.969 | 2.147 |
| M2-O2 | - | - | 2.053 |  |
| M1-O3 | - | 2.002 | - | - |
| M1-O4 | - | 2.016 | 2.124 |  |
| M2-O3 | - | - | 2.125 |  |
| M1-*Cl*1 | 2.406 | - | - | - |
| M1-*Cl*2 | 2.372 | - | - | - |
| Angles | [Pt(H_2_L)*Cl*_2_] | [Fe(HL)_2_] | [Zn_2_(HL)_2_]^2+^ | [Cd_2_(HL)_2_]^2+^ |
| N1-M1-O1 | - | - | 88.24 | 88.42 |
| N2-M1-O2 | 86.04 | 81.58 | - | - |
| N1-M1-O4 | - | - | 77.16 | 71.94 |
| N3-M1-O4 | - | 81.58 | - | - |
| O1-M1-O2 | - | - | - | 80.72 |
| O2-M1-O4 | - | 89.58 | 112.5 | 120.4 |
| O1-M2-O2 |  |  | 81.71 | 80.72 |
| O1-M2-O3 | - | - | 112.5 | 120.4 |
| O2-M2-N3 | - | - | 88.24 | 81.84 |
| N3-M2-O3 | - | - | 77.16 | 71.94 |
| N2-M1-N3 | - | 108.0 | - | - |
| O1-M1-N2 | - | 80.01 | - | - |
| O1-M1-N3 | - | 99.75 | - | - |
| O1-M1-O2 | - | 87.55 | 81.71 |  |
| O1-M1-O4 |  | 92.74 | - | - |
| O3-M1-N2 | - | 99.74 | - | - |
| O3-M1-N3 | - | 80.00 | - | - |
| O3-M1-O2 | - | 92.75 | - | - |
| O3-M1-O4 | - | 87.55 | - | - |
| *Cl*2-M1-N2 | 95.48 | - | - | - |
| *Cl*1-M1-O2 | 82.60 | - | - | - |
| *Cl*1-M1-*Cl2* | 95.88 | - | - | - |
| *Cl*2-M1-O2 | 178.4 | - | - | - |
| *Cl*1-M1-N2 | 168.6 | - | - | - |
| N1-M1O2 | - | - | 169.9 | 162.2 |
| O1-M1-O4 | - | - | 161.4 | 159.9 |
| N3-M2-O1 | - | - | 169.9 | 162.3 |
| O2-M2-O3 | - | - | 161.5 | 159.9 |
| N2-M1-O4 | - | 168.7 | - | - |
| N3-M1-O2 | - | 168.7 | - | - |
| O1-M1-O3 | - | 179.6 | - | - |
| N1-O1-O2-O4 | - | - | 7.801* | -4.113* |
| N3-O3-O1-O2 | - | - | 8.105* | 4.598* |
| N2-O2-O4-N3 | - | -7.901* | - | - |
| N2-O2-*Cl*1- *C2* | -0.117* | - | - | - |

*dihedral angle
